# NCK-dependent pericyte migration promotes pathological neovascularization in ischemic retinopathy

**DOI:** 10.1038/s41467-018-05926-7

**Published:** 2018-08-27

**Authors:** Alexandre Dubrac, Steffen E. Künzel, Sandrine H. Künzel, Jinyu Li, Rachana Radhamani Chandran, Kathleen Martin, Daniel M. Greif, Ralf H. Adams, Anne Eichmann

**Affiliations:** 1 Department of Internal Medicine, Yale Cardiovascular Research Center, Yale University School of Medicine, New Haven, CT 06511 USA; 2 Department of Tissue Morphogenesis and University of Münster, Faculty of Medicine, Max Planck Institute for Molecular Biomedicine, 48149 Münster, Germany; 3 INSERM U970, Paris Cardiovascular Research Center, 75015 Paris, France; 4 Department of Cellular and Molecular Physiology, Yale University School of Medicine, New Haven, CT 06520 USA

## Abstract

Pericytes are mural cells that surround capillaries and control angiogenesis and capillary barrier function. During sprouting angiogenesis, endothelial cell-derived platelet-derived growth factor-B (PDGF-B) regulates pericyte proliferation and migration via the platelet-derived growth factor receptor-β (PDGFRβ). PDGF-B overexpression has been associated with proliferative retinopathy, but the underlying mechanisms remain poorly understood. Here we show that abnormal, α-SMA-expressing pericytes cover angiogenic sprouts and pathological neovascular tufts (NVTs) in a mouse model of oxygen-induced retinopathy. Genetic lineage tracing demonstrates that pericytes acquire α-SMA expression during NVT formation. Pericyte depletion through inducible endothelial-specific knockout of Pdgf-b decreases NVT formation and impairs revascularization. Inactivation of the NCK1 and NCK2 adaptor proteins inhibits pericyte migration by preventing PDGF-B-induced phosphorylation of PDGFRβ at Y1009 and PAK activation. Loss of Nck1 and Nck2 in mural cells prevents NVT formation and vascular leakage and promotes revascularization, suggesting PDGFRβ-Y1009/NCK signaling as a potential target for the treatment of retinopathies.

## Abstract (web summary)

Pericytes are perivascular cells that regulate blood vessel formation and function. Here Dubrac et al. show that pericyte recruitment contributes to pathological neovascularisation in a mouse model of ischemic retinopathy, and that this depends on the regulation of PDGF-B signaling by NCK adaptor proteins.

## Introduction

Complications associated with neovascularization are the major cause of severe vision loss in patients with the wet form of age-related macular degeneration (AMD), proliferative diabetic retinopathy (PDR), and retinopathy of prematurity (ROP). PDR and ROP are characterized by chronic ischemia that drives formation of NVTs, which are clusters of convoluted capillary loops exhibiting excessive endothelial cell (EC) proliferation and bleeding^1–3^. Aberrant neovascularization induces vitreous hemorrhage and macular edema, ultimately leading to visual impairment and blindness.

The major therapeutic challenge is to block NVT formation while simultaneously improving retinal revascularization and healing. Chronic ischemia increases the expression of growth factors such as vascular endothelial growth factor-A (VEGF-A) and PDGF-B^2,3^. Retinal neoangiogenesis and VEGF-A inhibition are the primary targets to treat retinal vascular diseases. Intravitreal VEGF-A blockers are currently undergoing clinical trials for patients with ROP and certain cases of PDR, with positive effects^4–8^ (https://clinicaltrials.gov/ct2/results?cond=PDR&term=vegf&cntry=&state=&city=&dist=). However, prolonged VEGF-A inhibition has been associated with neuronal toxicity and ocular complications^9–11^. Therefore, it is crucial to identify additional therapeutic targets.

Pericytes are perivascular cells that regulate vessel growth, maturation, and permeability^12^. The mouse retinal vasculature develops after birth and extends from the optic nerve to the periphery in an organized, branched network led by endothelial tip cells^12,13^. Among various factors, tip cells release PDGF-B, which binds to platelet-derived growth factor receptor β (PDGFRβ) on pericytes and induces their recruitment to the nascent sprout. Pericytes cover the tip cell at its connection to the follower stalk cell. In this position, they spatially restrain VEGF-A activity through soluble VEGFR1, leaving the angiogenic end of the tip free to extend filopodia^14^. Pericytes stabilize sprouts^15,16^ and contribute to the forming blood–retinal barrier (BRB) that becomes fully functional at P10 and provides a homeostatic environment for proper neural function^17–19^. The PDGF-B/PDGFRβ signaling pathway is also essential to recruit pericytes to growing brain vessels and for formation of the blood–brain barrier (BBB)^12^. Whether developmental and adult brain angiogenesis and BBB formation occur through the same mechanisms remains to be defined.

Pericytes have long been implicated in the initiation and the progression of diabetic retinopathy (DR)^20^. Several studies proposed that pericyte detachment and loss of BRB integrity lead to increased permeability and macular edema, which precede PDR^20–23^. Targeting PDGFs has been proposed as a potential therapeutic option in wet AMD^24^. However, a PDGF antagonist failed to show improvement in best-corrected visual acuity in combination treatment over standard anti-VEGF monotherapy in phase III studies (https://clinicaltrials.gov/ct2/show/NCT01944839?term=fovista&rank=3). Here, we investigated the PDGFRβ downstream signaling pathways involved in pericyte recruitment, attachment, and survival in an oxygen-induced retinopathy (OIR) model in mice that mimics the vascular defects of human ROP and certain aspects of PDR^1,25,26^.

PDGFRβ is widely expressed on the surface of pericytes and required for pericyte migration, proliferation, and survival^12,16^. After ligand binding and receptor dimerization, phosphorylated tyrosines in the PDGFRβ intracellular domain recruit scaffold proteins to induce several signaling pathways^27^. Among those, we show that the NCK1 and NCK2 adaptor proteins are selectively required for PDGF-B-induced pericyte migration and recruitment to sprouting endothelial cells. In mammals, *Nck1* and *Nck2* have broad and overlapping expression patterns and function redundantly^28,29^. NCK1/2 act as adapters by linking receptor tyrosine kinases to downstream signaling networks. NCK1/2 interact with the p21-activated kinase (PAK) family of serine/threonine kinases and their upstream activators, RAC1/CDC42, to regulate cytoskeletal dynamics^28,30^. In fibroblasts, NCKs bind to phosphorylated Tyr-751 and Tyr-1009 of PDGFRβ allowing the activation of PAK, CDC42, and migration in vitro^31^. The role of NCK1/2 in pericyte biology was unknown.

In this study, we show that ischemic retinopathy NVTs are formed by pathological pericyte activation and dysfunction, which contributes to defective revascularization, vascular leak, and hemorrhage. We identify NCK1/2 as an essential component of the PDGF-B/PDGFRβ signaling machinery that drives pericyte migration in vitro and in vivo, and show that mural cell-specific *Nck* deletion inhibits NVT formation. Our findings demonstrate that selectively targeting pericyte recruitment inhibits developmental and pathological neovascularization, identifying PDGFRβ-NCK1/2 signaling as a novel therapeutic target.

## Results

### Characterization of mural cells in ischemic retinopathy

**Fig. 1:**
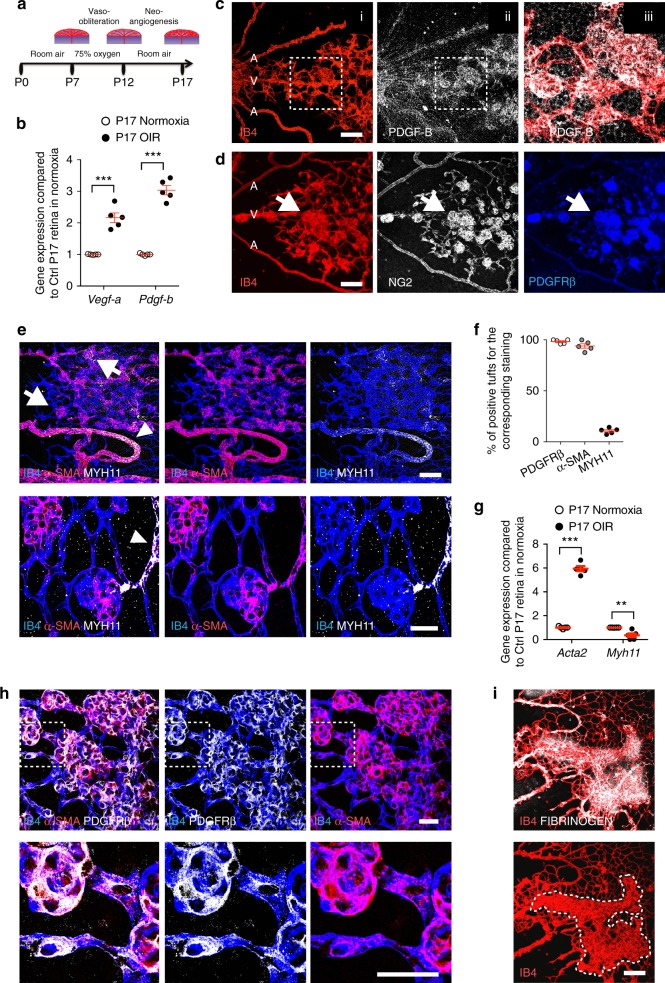
Abnormal mural cells cover NVTs in the OIR retina. **a** Schematic of the OIR model. **b** qPCR analysis of *Vegf-a* and *Pdgf-b* expression in P17 OIR retinas compared to P17 retinas in normoxia (*n* = 5 mice). Error bars: s.e.m. ^⋆^^⋆^^⋆^*P* < 0.001, two-way ANOVA Sidak’s multiple comparisons test. **c** IB4 (i) and PDGF-B (ii) double staining of retina from P17 OIR mice and magnified area (iii) of boxed area in ii. **d** IB4, NG2, and PDGFRβ triple staining shows tufts expressing pericyte markers (white arrows) in P17 OIR retinas. **e** IB4, α-SMA, and MYH11 triple staining of P17 OIR retinas. Tufts are covered with MYH11− SMA+ cells (arrows). Note that arterioles surrounding the NVTs are covered with MYH11+ SMA+ cells (arrowhead). (Bottom) Higher magnifications of tufts, note labeling with α-SMA but not MYH11. **f** Quantification of tufts labeled with the indicated markers in P17 OIR retinas (*n* = 5 mice, error bars: s.e.m.). **g** qPCR analysis of *Acta2* (encoding α-SMA) and *Myh11* expression in P17 OIR retinas compared to P17 retinas in normoxia (*n* = 5 mice). Error bars: s.e.m. ^⋆^^⋆^^⋆^*P* < 0.001, ^⋆^^⋆^*P* < 0.01, two-way ANOVA Sidak’s multiple comparisons test. **h** Top, IB4, α-SMA, and PDGFRβ triple staining of vascular tufts from P17 OIR retinas. (Bottom) Higher magnification of the boxed areas. **i** IB4 and FIBRINOGEN double staining of vascular tufts from P17 retinas in OIR. The dotted line marks the tufts. Scale bars, 100 μm (**c**–**e** top, **i**) and 30 μm (**e** bottom, **h**)

**Fig. 2:**
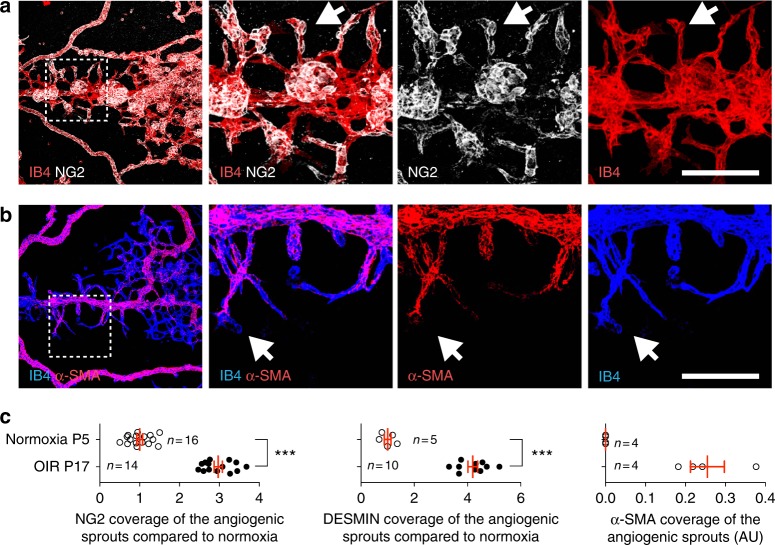
Excessive mural cell covering of angiogenic sprouts in the OIR retina. **a** IB4 and NG2 double staining of retina from P17 OIR mice and magnification of boxed area. Tip cells are covered with NG2+ pericytes (arrows). **b** IB4 and α-SMA double staining of P17 OIR retinas and magnification of boxed area. Sprouts are covered with α-SMA+ mural cells (arrows). **c** Quantification of angiogenic sprouts labeled with NG2, DESMIN, and α-SMA in P5 normoxia and OIR retinas. Number of retinas used for quantification is indicated. Error bars: s.e.m. ^⋆^^⋆^^⋆^*P* < 0.001, Mann–Whitney *U* test. Scale bars, 100 μm

To study the contribution of pericytes to ocular neovascular disease, we used the OIR mouse model. Hyperoxia (75% oxygen) exposure of neonatal mice between postnatal day (P) 7 and P12 triggers vaso-obliteration and a capillary-free avascular area in the center of the retina, which becomes ischemic after return to room air (Fig. 1a). Hypoxic cells secrete excessive amounts of angiogenic factors, which lead to pathological sprouting and formation of abnormal NVTs that are leaky and prone to bleeding. P15 and P17 OIR retinas indeed showed an increase of *Vegf-a* and *Pdgf-b* expression compared to normal P15 and P17 retinas (Fig. 1b and Supplementary Fig. 1a). Using immunostaining, we found that PDGF-B is robustly expressed by ECs in pathologically expanding NVTs (Fig. 1c). Antibody staining with mural cell markers neural/glial antigen 2 (NG2), PDGFRβ, DESMIN, α-smooth muscle actin (α-SMA), and the smooth muscle marker myosin 11 (MYH11) showed that mural cells covering the tufts were highly positive for NG2, DESMIN, PDGFRβ, and α-SMA, but negative for MYH11 (Fig. 1d–g and Supplementary Fig. 1b, c). More than 95% of the tufts were PDGFRβ+ and α-SMA+, while only 10% were weakly MYH11+ (Fig. 1f). Quantitative polymerase chain reaction (qPCR) analysis confirmed that *Acta2* (encoding α-SMA) expression increased and *Myh11* expression decreased in P17 OIR retina compared to normoxic retinas (Fig. 1g). Double staining of PDGFRβ and α-SMA revealed that the mural cells covering the NVTs were PDGFRβ+ and α-SMA+ double positive (Fig. 1h). In contrast, developing retinas at P5 and P12 had NG2+ PDGFRβ+, and DESMIN+ triple-positive and α-SMA− and MYH11− pericytes covering capillaries, whereas smooth muscle cells (SMCs) were positive for all markers and restricted to arterioles (Supplementary Fig. 2 and Supplementary Fig. 3a, b). At later stages, P17 and P25 adult retinas revealed MYH11+ but α-SMA− pericytes covering capillaries (Supplementary Fig. 3–f). Immunostaining showed accumulation of the basement membrane (BM) components fibronectin, collagen4, and laminin on the NVTs (Supplementary Fig. 4a). Moreover, staining of the vascular permeability marker FIBRINOGEN showed abundant leakage co-localized with NVTs compared to the P17 normoxic retina of (Fig. 1i and Supplementary Fig. 4b–d). Strikingly, the OIR sprouting capillary tips were covered by NG2+ DESMIN+ mural cells, compared to the tip cells of P5 normoxic retinas that were not pericyte covered at their leading edge (Fig. 2a, c and Supplementary Fig. 2a, c). Furthermore, the abnormal OIR pericytes covering tip cells were α-SMA+ (Fig. 2b, c and Supplementary Fig. 2b). These data show that in OIR, NVTs and angiogenic sprouting tips recruit excessive mural cells that are different from normal pericytes and SMCs.

### Origin of the abnormal mural cells in ischemic retinopathy

**Fig. 3:**
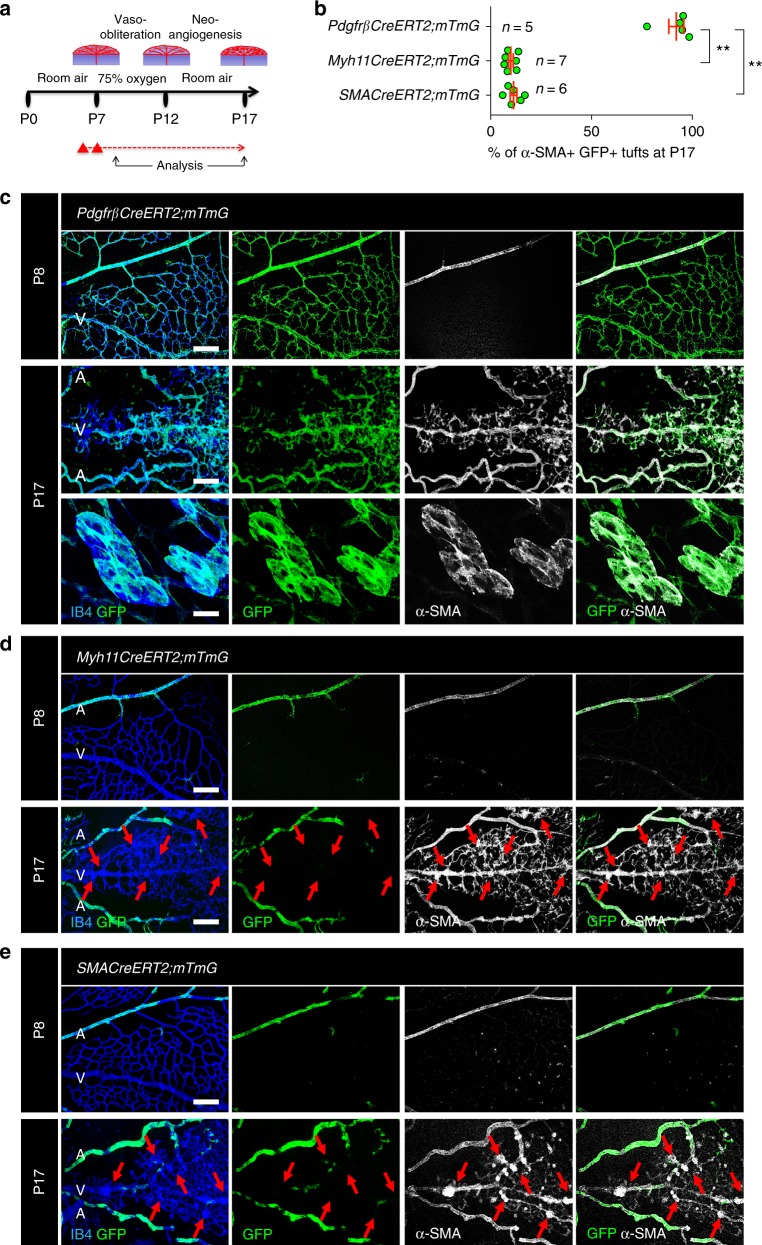
Mural cells covering tufts are derived from PDGFRβ-positive pericytes. **a** Strategy for genetic labeling of mural cells by tamoxifen treatment (red triangles) in the corresponding *CreERT2;mTmG* mice. **b** Quantification of tufts labeled with α-SMA and GFP in OIR retinas from the indicated P17 mice. The number of retinas used for quantification is indicated. Error bars: s.e.m. ^⋆^^⋆^*P* < 0.01, Mann–Whitney *U* test. **c** IB4, α-SMA, and GFP triple staining of retinas from P8 and P17 *Pdgfrβ-CreERT2;mTmG* mice. **d** IB4, α-SMA, and GFP triple staining of retinas from P8 and P17 *Myh11CreERT2;mTmG* mice. **e** IB4, α-SMA, and GFP triple staining of retinas from P8 and P17 *SMACreERT2;mTmG* mice. Red arrows in **d** and **e** show α-SMA+GFP− NVTs. Scale bars, 100 μm (**c** top and middle, **d**, **e**) and 60 μm (**c** bottom)

The abnormal mural cells in the OIR retina could be derived from α-SMA− pericytes, or from α-SMA+ SMCs. To distinguish between these possibilities, we used *PdgfrβCreERT2*, *Myh11CreERT2*, and *SMACreERT2* mice interbred with *Rosa26-mTmG* Cre reporter mice to genetically label the mural cells and their descendants. Tamoxifen injection at P6 and P7 activated green fluorescent protein (GFP) expression in Cre-expressing cells and their progeny with a very high efficiency (Fig. 3a and Supplementary Fig. 5a). One day later, *PdgfrβCreERT2*-mediated recombination labeled both pericytes on capillaries and α-SMA+ SMCs covering the arterioles (Fig. 3c). In P17 OIR *PdgfrbCreERT2;mTmG* retinas, more than 95% of the α-SMA+ tufts were covered with GFP+ cells (Fig. 3b, c). In P8 *Myh11CreERT2;mTmG* and *SMACreERT2;mTmG* retinas, GFP expression was limited to α-SMA+ SMCs covering arterioles (Fig. 3d, e). In P17 OIR retinas, we found that <10% of the α-SMA+ tufts were GFP+-labeled descendants of GFP+ SMCs (Fig. 3b, d, e). When mural cells were labeled during the hypoxia period by tamoxifen at P12, P13, and P14, α-SMA+ tufts also showed GFP expression in *SMACreERT2;mTmG*, but not *Myh11CreERT2;mTmG*, mice (Supplementary Fig. 5b). These data show that NVT-associated PDGFRβ+ α-SMA+ MYH11− cells are derived from PDGFRβ+ α-SMA− MYH11− pericytes.

### Endothelial PDGF-B is required for pericyte coverage and retinal angiogenesis

**Fig. 4:**
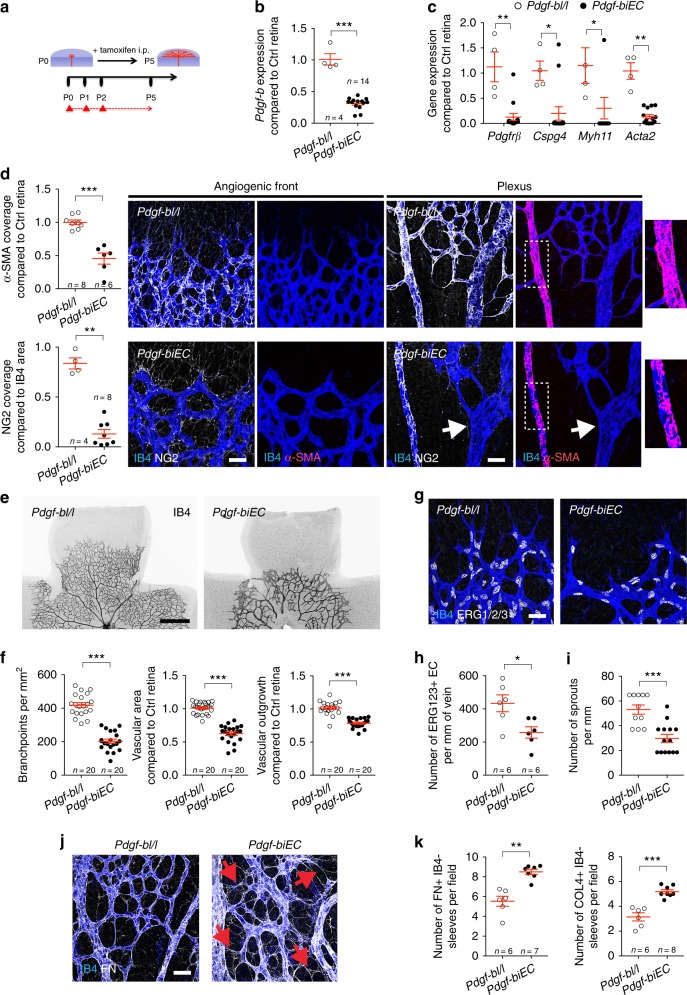
Postnatal endothelial *Pdgf-b* deletion induces vascular defects. **a** Experimental strategy to assess retinal vasculature development (P0–P5). The red triangles indicate tamoxifen injections. **b** qPCR measurement of *Pdgf-b* levels in mouse retina. Number of retinas used for quantification is indicated. Error bars represent s.e.m. ^⋆^^⋆^^⋆^*P* < 0.001, Mann–Whitney *U* test. **c** qPCR analysis of *Pdgfrβ*, *Cspg4* (encoding NG2), *Myh11*, and *Acta2* (encoding α-SMA) expression in P5 mouse retina. *N* = 4 *Pdgf-bl/l* and *N* = 14 *Pdgf-biEC* retinas. Error bars represent s.e.m. ^⋆^*P* < 0.05; ^⋆^^⋆^*P* < 0.01, two-way ANOVA Sidak’s multiple comparisons test. **d** Left, quantification of α-SMA (top) and NG2 (bottom) coverage of IB4+ vessels of P5 retina. Number of retinas used for quantification is indicated. Error bars represent s.e.m. ^⋆^^⋆^*P* < 0.01; ^⋆^^⋆^^⋆^*P* < 0.001, Mann–Whitney *U* test. (Right) IB4, NG2, and α-SMA staining of the angiogenic front and the plexus of P5 retina. Note microaneurysm formation in *Pdgf-biEC* mice (arrow). Higher magnification of α-SMA staining in boxed areas is shown. **e** IB4 staining of retinal flat mounts of P5 mice (negative images of the fluorescent signal). **f** Quantification of branchpoints, vascular area, and vascular outgrowth. Number of retinas used for quantification is indicated. Error bars represent s.e.m. ^⋆^^⋆^^⋆^*P* < 0.001, Mann–Whitney *U* test. **g** IB4, ERG1/2/3 double staining of the angiogenic front of P5 retina. **h** Quantification the ERG1/2/3+ EC in the venule of P5 retina. Number of retinas used for quantification is indicated. Error bars represent s.e.m. ^⋆^*P* < 0.05, Mann–Whitney *U* test. **i** Quantification of sprouts in the angiogenic front (*n* = 11 imaging fields for *Pdgf-bl/l* from four retinas and *n* = 14 for *Pdgf-biEC* from four retinas). Error bars represent s.e.m. ^⋆^^⋆^^⋆^*P* < 0.001, Mann–Whitney *U* test. **j** IB4 and fibronectin (FN) staining of P5 retina. Red arrows show empty BM sleeves of retracting vessels. **k** Quantification of FN+ IB4− and COL4+ IB4− sleeves per ×63 pictures. Number of retinas used for quantification is indicated. Error bars represent s.e.m. ^⋆^^⋆^*P* < 0.01, ^⋆^^⋆^^⋆^*P* < 0.001, Mann–Whitney *U* test. Scale bars, 30 μm (**d**, **g**, **j**) and 500 μm (**e**)

We next tested if *Pdgf-b* was required for pericyte recruitment and angiogenesis in developing retinas. Because ECs are the main source of PDGF-B in the retina^32^, we generated inducible, endothelial-specific *Pdgf-b* deletions by interbreeding *Pdgf-b* lox mice^32^ with the *Cdh5-CreERT2* mice (hereafter referred to as *Pdgf-biEC*). Tamoxifen was injected at birth (P0/1/2) and retinas were analyzed at P3 and P5 (Fig. 4a, Supplementary Fig. 6a). We confirmed the efficiency of *Pdgf-b* deletion by qPCR (Fig. 4b and Supplementary Fig. 6b). Twenty-four hours after the last tamoxifen injection, *Pdgf-biEC* retinas showed more than 50% decrease of pericyte coverage (Supplementary Fig. 6c, d) and minor vascular defects (Supplementary Fig. 6e, f) compared to tamoxifen-injected, *Cre*-negative control littermates. At P5, the expression of pericyte and SMC genes was strongly decreased in *Pdgf-biEC* retinas (Fig. 4c). NG2 and α-SMA staining showed an almost complete inhibition of pericyte and SMC coverage in the absence of PDGF-B (Fig. 4d). Vessel dilations and micro-aneurisms were frequently observed (Fig. 4d and Supplementary Fig. 6g). Staining of the retinal vasculature with isolectin-B4 (IB4) showed that vessel area, branching, and vascular radial expansion were severely impaired in the absence of pericytes (Fig. 4e, f). These data confirm that pericytes are essential for retinal angiogenesis^16,32^. During postnatal development of the retinal vasculature, endothelial cell proliferation is restricted to the venules and the vascular front. The quantification of the ERG123+ endothelial cells^33^ in the venule and in the vascular front revealed lower cell densities in *Pdgf-biEC* than in littermate controls (Fig. 4g, h and Supplementary Fig. 6h). The number of angiogenic sprouts at the vascular front of *Pdgf-biEC* retinas was decreased in comparison with control littermates (Fig. 4g, i). Moreover, pericyte depletion strongly impaired vessel stability, as shown by an increase of COLLAGEN 4+ or FIBRONECTIN+, IB4-negative empty BM sleeves, indicative of active vessel regression in *Pdgf-biEC* retinas (Fig. 4j, k and Supplementary Fig. 6i). Taken together, these data show that endothelial PDGF-B is required for pericyte recruitment and promotes postnatal sprouting angiogenesis in vivo.

### Endothelial *Pdgf-b* promotes NVT formation in ischemic retinopathy

**Fig. 5:**
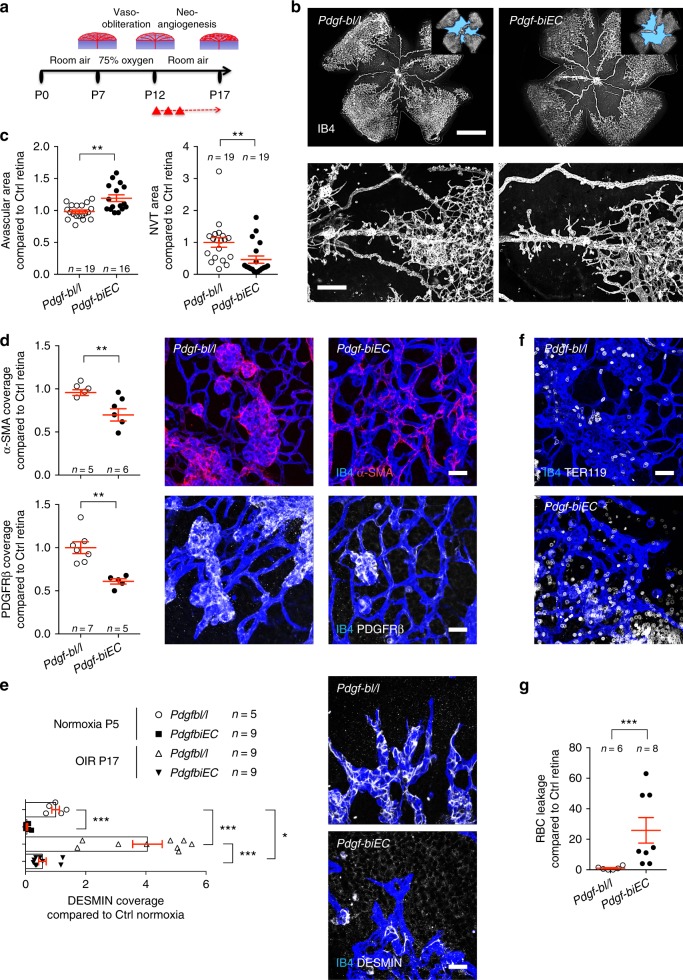
Endothelial PDGF-B promotes tuft formation and OIR revascularization. **a** Strategy to assess neoangiogenesis after OIR. The triangles indicate tamoxifen injection. **b** Retinal flat mounts after OIR. Insets show avascular area measured for quantification. Lower panels show higher-magnification images of pathological vascular tufts. **c** Avascular area (left) and NVT (right) quantification. Number of retinas used for quantification is indicated. Error bars represent s.e.m. ^⋆^^⋆^*P* < 0.01, Mann–Whitney U test. **d** (Left) Quantification of α-SMA (top) and PDGFRβ (bottom) coverage of IB4+ vascular tufts. (Right) IB4 and α-SMA (top) or IB4 and PDGFRβ (bottom) double staining of the NVTs. Number of retinas used for quantification is indicated. Error bars represent s.e.m. ^⋆^^⋆^*P* < 0.01, Mann–Whitney *U* test. **e** (Left) Quantification of DESMIN coverage of IB4+ vascular sprouts. (Right) IB4 and DESMIN double staining of the angiogenic sprouts in P5 normoxic and P17 OIR retinas. Number of retinas used for quantification is indicated. Error bars represent s.e.m. ^⋆^^⋆^*P* < 0.01, Mann–Whitney *U* test. **f**, **g** Images (**f**) and quantification (**g**) of TER119+ RBC leakage in *Pdgf-biEC* compared to control littermate mice. Number of mice used for quantification is indicated. Error bars represent s.e.m. ^⋆^^⋆^^⋆^*P* < 0.001, Mann–Whitney *U* test. Scale bars, 1 mm (**b** top), 100 μm (**b** bottom), and 30 μm (**d**–**f**)

To determine whether PDGF-B-dependent pericyte recruitment contributed to NVT formation in ischemic retinopathy, we subjected *Pdgf-biEC* mice to OIR. Induction of *Pdgf-b* deletion was done after hyperoxia exposure during the neovascularization period (P12–P17; Fig. 5a). As expected, *Pdgf-bl/l* control littermates and *Pdgf-biEC* mice displayed similar avascular area prior to gene deletion at P12 (Supplementary Fig. 7a). At P17, *Pdgf-biEC* mice showed reduced NVT formation and revascularization (Fig. 5b, c). qPCR analysis of P15 OIR retinas revealed only 60% decrease of *Pdgf-b* expression in the *Pdgf-biEC* mice, suggesting the existence of other cell sources of PDGF-B besides ECs^34,35^ (Supplementary Fig. 7b). Despite incomplete *Pdgf-b* deletion, the expression of pericyte and SMC genes was decreased in *Pdgf-biEC* retinas, while *Vegf-a* expression was similar to controls (Supplementary Fig. 7b). Staining with antibodies against α-SMA, PDGFRβ, and DESMIN confirmed that *Pdgf-b* deletion strongly decreased mural cell coverage of the NVTs and of the angiogenic sprouts (Fig. 5d, e and Supplementary Fig. 7c). As previously described, pericyte depletion also increased other vascular defects including capillary dilations and TER119+ red blood cell (RBC) leakage^19,36^ (Fig. 5f and g and Supplementary Fig. 7d). These results show that blockade of endothelial PDGF-B-dependent pericyte recruitment inhibited pathological NVT and vessel regrowth, albeit with concomitant adverse effects on blood vessels.

### NCK1 and NCK2 are required for PDGF-B-induced pericyte migration in vitro

**Fig. 6:**
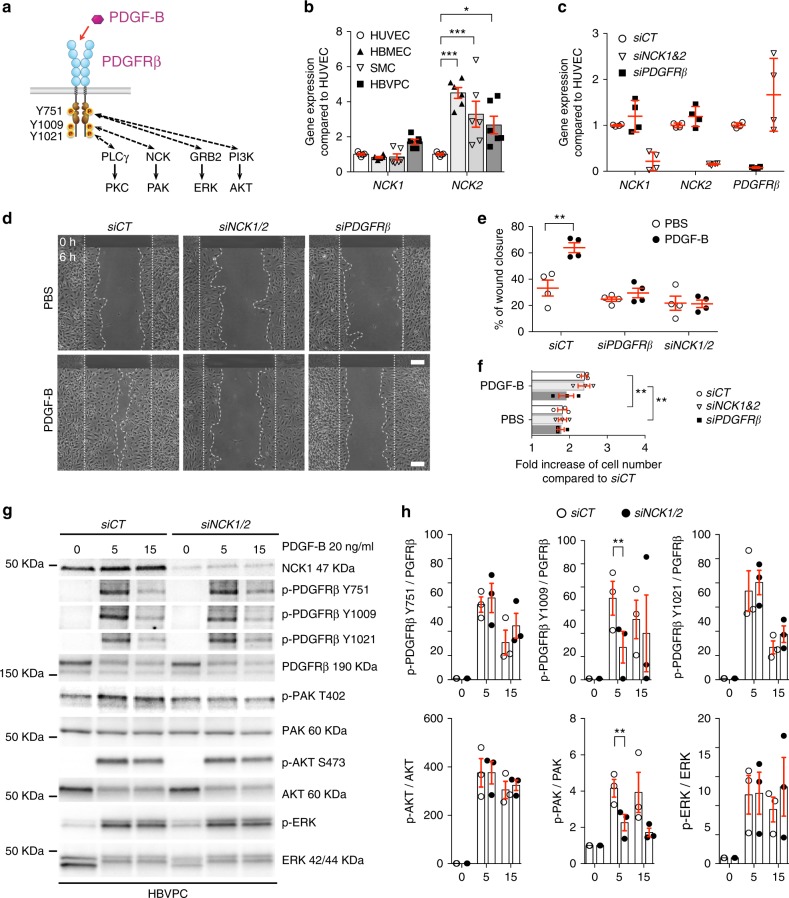
NCK is essential for PDGF-B-induced pericyte migration in vitro. **a** Schematic of PDGFRβ downstream signaling. NCK adaptor proteins bind the phospho-Y1009 on the PDGFRβ receptor to regulate PDGF-B-induced PAK activation, while other downstream effectors bind to different phosphosites. **b** qPCR measurement of *NCK1* and *NCK2* levels in HBMEC (*n* = 6), human brain SMC (*n* = 6), and HBVPC (*n* = 6) compared to HUVEC (*n* = 5). Error bars represent s.e.m. ^⋆^*P* < 0.05, ^⋆^^⋆^^⋆^*P* < 0.001, two-way ANOVA with Sidak’s multiple comparisons test. **c** qPCR analysis of *NCK1*, *NCK2*, and *PDGFRβ* expression in HBVPC transfected with siRNAs against *NCK1* and *NCK2* or *PDGFRβ* compared to control siRNA (siCT) (*n* = 4 experiments). Error bars represent s.e.m. **d** Scratch-wound migration assay using HBVPC treated with siRNAs and stimulated with recombinant proteins as indicated for 6 h. Top panels show the wound edges at 0 h. Dashed lines mark wound migration edges at 0 h (straight lines) and 6 h. **e** Quantification of wound closure (*n* = 4 experiments). Error bars represent s.e.m. ^⋆^^⋆^*P* < 0.01, Student’s *t* test. **f** Proliferation assay using HBVPC transfected with control (siCT), *NCK1* and *NCK2* or *PDGFRβ* siRNAs and stimulated with PDGF-B (*n* = 3 experiments). Error bars represent s.e.m. ^⋆^^⋆^*P* < 0.01, Student’s *t* test. **g** Western blot using HBVPC stimulated with PDGF-B and probed with the indicated antibodies. **h** Quantifications of blots shown in **g** (*n* = 3 experiments). Error bars represent s.e.m. ^⋆^^⋆^*P* < 0.01, Student’s *t* test. Scale bars, 150 μm (**d**)

We next reasoned that targeting specific PDGFRβ downstream signaling pathways might prevent NVT formation without deleterious effects on angiogenesis. The two closely related SH2/SH3 adapter proteins, NCK1 and NCK2, bind to phosphorylated Tyr-751 and Tyr-1009, respectively, in the activated PDGFRβ to promote fibroblast migration^31,37^. We hypothesized that NCK1 and NCK2 regulated PDGF-B**-**induced pericyte migration through PAK activation (Fig. 6a). We first tested the effects of NCK1 and NCK2 in human brain vascular pericyte (HBVPC) in vitro. HBVPCs grown in endothelial cell medium EGM2 expressed high level of pericyte markers NG2, PDGFRβ, but were negative for SMC markers or markers of ECs (CD31)^38^ (Supplementary Fig. 8a, b). HBVPCs show similar *NCK1* and *NCK2* expression compared to brain EC and SMC, and combination of *NCK1* and *NCK2* small interfering RNAs (siRNAs) efficiently knocked down *NCK1* and *NCK2* in HBVPC (Fig. 6b, c). *NCK1* and *NCK2* knockdown abolished PDGF-B-induced HBVPC migration (Fig. 6d, e), but not proliferation (Fig. 6f). As PDGF-B-induced cell migration requires the activation of PAK^27,39^, we asked whether PAK was activated downstream of NCK1 and NCK2 in response to PDGFRβ activation. PDGF-B induced PDGFRβ phosphorylation, extracellular signal-regulated kinases (ERKs), serine/threonine protein kinase AKT, and PAK phosphorylation in HBVPC transfected with control siRNA (Fig. 6g). *NCK1* and *NCK2* siRNA did not affect PDGFRβ expression (Fig. 6c, g and Supplementary Fig. 8c), but PDGF-B-induced phosphorylation of PAK was strongly reduced by *NCK1* and *NCK2* knockdowns (Fig. 6g, h). Combined *NCK1* and *NCK2* deletions also reduced PDGFRβ Y1009 phosphorylation following PDGF-B treatment, but did not affect the activation of PDGFRβ Y751, Y1021, AKT, and ERK phosphorylation (Fig. 6g, h). Taken together, these results demonstrate that NCK1 and NCK2 are required for PDGF-B-induced PAK activation and pericyte migration.

### NCK1 and NCK2 promote pericyte migration and retinal vascularization

**Fig. 7:**
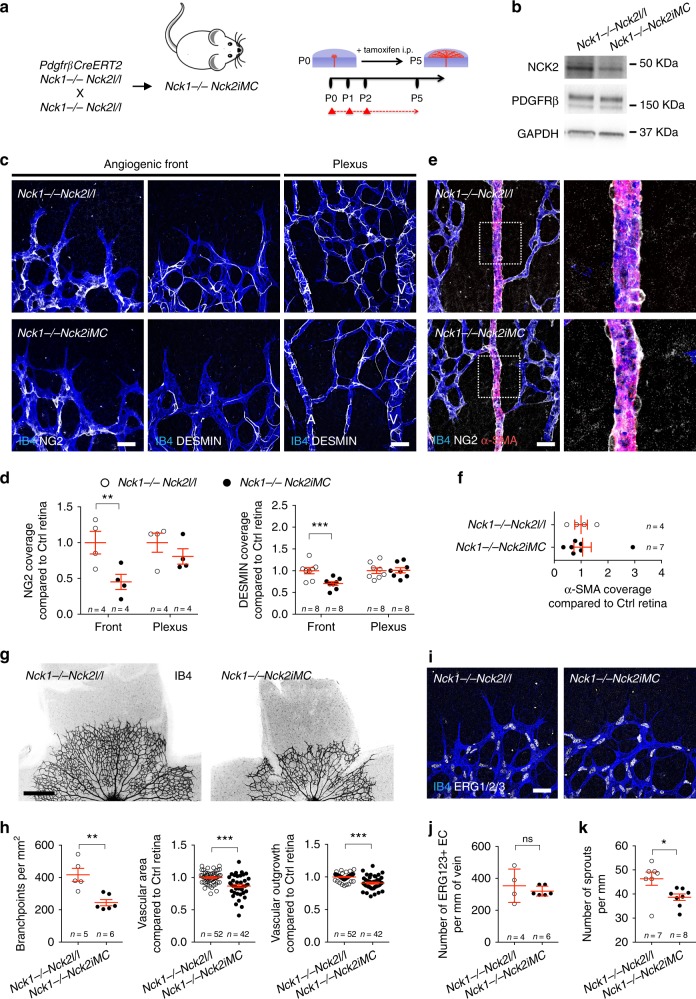
NCK promotes pericyte migration and retinal vascularization. **a** Experimental strategy to delete *Nck1* and *Nck2* in pericytes during retinal development (P0–P5). The red triangles indicate tamoxifen injections. **b** Western blot against NCK2, PDGFRβ, and GAPDH using P5 total lung lysate from P0/1/2 tamoxifen-injected mice. **c** IB4, NG2 (left), and IB4, DESMIN (middle) double staining of the angiogenic front and IB4, DESMIN double staining of the plexus (right) of P5 retina. **d** Quantification of NG2 (left) and DESMIN (right) coverage of IB4+ vessels of the angiogenic front and the plexus of P5 retina. Note the specific decrease of pericyte coverage in the angiogenic front of *Nck1−/−Nck2iMC* double-mutant mice. Number of retinas used for quantification is indicated. Error bars represent s.e.m. ^⋆^^⋆^^⋆^*P* < 0.001, Mann–Whitney *U* test. **e** (Left) IB4, NG2, and α-SMA triple staining of P5 retina. (Right) Higher magnification of the boxed areas. **f** Quantification of α-SMA coverage. Number of retinas used for quantification is indicated. Error bars represent s.e.m. **g** IB4 staining of retinal flat mounts of P5 mice with the indicated genotypes (negative images of the fluorescent signal). **h** Quantification of branchpoints, vascular area, and vascular outgrowth. Number of retinas used for quantification is indicated. Error bars represent s.e.m. ^⋆^^⋆^*P* < 0.01, ^⋆^^⋆^^⋆^^⋆^*P* < 0.001, Mann–Whitney *U* test. **i** IB4, ERG1/2/3 double staining of the angiogenic front of P5 retina with the indicated genotype. **j** Quantification the ERG1/2/3-positive EC. Number of retinas used for quantification is indicated. Error bars represent s.e.m. NS: non-significant. Mann–Whitney *U* test. **k** Quantification of sprouts in the angiogenic front. Number of retinas used for quantification is indicated. Error bars represent s.e.m. ^⋆^^⋆^*P* < 0.01, Mann–Whitney *U* test. Scale bars, 30 μm (**c**, **e**, **i**) and 500 μm (**g**)

We next determined whether *Nck1* and *Nck2* were required for pericyte recruitment and vessel coverage during postnatal retina development. Because *Nck1* knockout mice did not show vascular and mural cell defects^29^, we deleted *Nck2* in mural cells on a *Nck1*-null background by intercrossing *Nck1−/−Nck2l/l* mice with *PdgfrβCreERT2* mice. Because this *Cre* line deletes in pericytes and SMCs, we named the resulting mutants *Nck1−/−Nck2iMC* (for induced in mural cells) (Fig. 7a). Western blot using total lysate from P5 lungs or retinas of *Nck1−/−Nck2iMC* double-knockout mice showed reduced expression of NCK2 after CRE activation by tamoxifen (P0/1/2) (Fig. 7b and Supplementary Fig. 9a, b). The combined loss of NCK1 and NCK2 did not affect PDGFRβ expression, but we observed a 25% decrease in PDGFRβ Y751 and a 50% decrease in PDGFRβ Y1009 phosphorylation (Supplementary Fig. 9a, b). We next examined pericyte coverage by staining with NG2 and DESMIN. While pericyte coverage of the vascular plexus was comparable to control *Nck1−/−Nck2l/l* littermates at P5, deletion of *Nck1*and *Nck2* significantly decreased pericyte recruitment at the angiogenic front (Fig. 7c, d and Supplementary Fig. 9c, d). α-SMA staining revealed that SMC coverage was not affected (Fig. 7e, f). IB4 staining of P5 retinas showed that the number of branches, the vessel area, and vessel outgrowth were significantly reduced in *Nck1−/−Nck2iMC* mice (Fig. 7g, h). At P12 however, the mutant mice showed normal vascularization of the superficial retinal layer and only slightly impaired formation of the deeper vascular retinal layers, suggesting that the effect of mural cell *Nck1* and *Nck2* deletion on retinal vascular development was likely transient (Supplementary Fig. 9e). While the EC density was not affected by loss of NCK1 and NCK2, the number of angiogenic sprouts was decreased in *Nck1−/−Nck2iMC* retinas at P5 (Fig. 7i–k and Supplementary Fig. 10a). Moreover, we measured a mild but significant change on vessel diameter in the retina of the mutant mice (Supplementary Fig. 10b). Together, these data show that NCK1 and NCK2 are required for pericyte migration and coverage of new growing capillary sprouts.

### Murall cell NCK1 and NCK2 is required for neovascularization in OIR

**Fig. 8:**
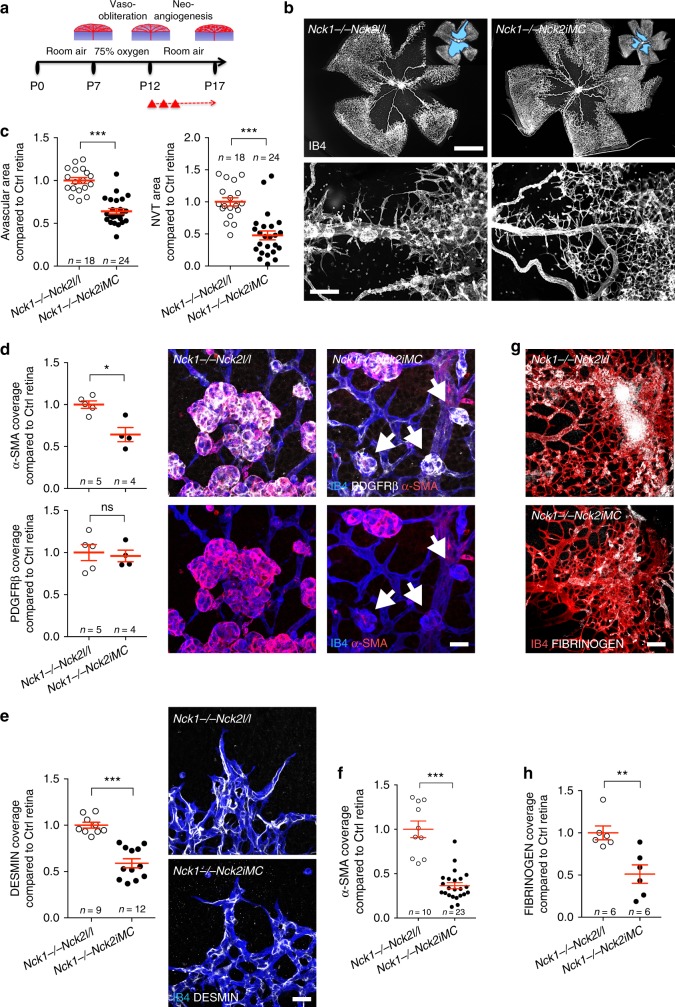
Pericyte *Nck1*/*2* deletion improves OIR revascularization. **a** Strategy to assess neoangiogenesis after OIR. *Nck2* deletion is induced at P12, P13, and P14. **b** Flat mounts of P17 *Nck1−/−Nck2iMC* and littermate control OIR retinas. Insets show avascular area measured for quantification. Lower panels show higher-magnification images of pathological vascular tufts. **c** Avascular area (top) and NVT (bottom) quantification. Number of retinas used for quantification is indicated. Error bars represent s.e.m. ^⋆^^⋆^^⋆^*P* < 0.001, Mann–Whitney *U* test. **d** (Left) Quantification of α-SMA (top) and PDGFRβ (bottom) coverage of IB4+ vascular tufts. Number of retinas used for quantification is indicated. Error bars represent s.e.m. NS: non-significant. ^⋆^*P* < 0.05, Mann–Whitney *U* test. (Right) IB4, PDGFRβ, and α-SMA triple staining of the NVTs. Note the smaller and sometimes α-SMA-negative tufts in *Nck1−/−Nck2iMC* double-mutant mice (arrows). **e** (Left) Quantification of DESMIN coverage of IB4+ vascular sprouts in P17 OIR retinas (*n* = 9 imaging fields for *Nck1−/−Nck2l/l* from 6 retinas and *n* = 12 for *Nck1−/−Nck2iMC* from 6 retinas). (Right) IB4 and DESMIN double staining of the angiogenic sprouts. Error bars represent s.e.m. ^⋆^^⋆^^⋆^*P* < 0.001, Mann–Whitney *U* test. **f** Quantification of α-SMA coverage of IB4+ vascular sprouts in P17 OIR retinas (*n* = 10 imaging fields for *Nck1−/−Nck2l/l* from 2 retinas and *n* = 23 for *Nck1-/-Nck2iMC* from 4 retinas). Error bars represent s.e.m. ^⋆^^⋆^^⋆^*P* < 0.001, Mann–Whitney *U* test. **g**, **h** Images (**g**) and quantification (**h**) of FIBRINOGEN leakage in *Nck1−/−Nck2iMC* compared to control littermate mice. Number of mice used for quantification is indicated. Error bars represent s.e.m. ^⋆^^⋆^*P* < 0.01, Mann–Whitney *U* test. Scale bars, 1 mm (**b** top), 100 μm (**b** bottom, **g**), and 30 μm (**d**, **e**)

To explore the function of mural cell NCK in pathological angiogenesis, we induced ischemic retinopathy in *Nck1−/−Nck2iMC* mice. Induction of *Nck2* deletion was done after exposure to hyperoxia, during the neovascularization period (P12–P17; Fig. 8a). As expected, the P12 avascular area was similar between *Nck1−/−Nck2l/l* control littermates and *Nck1−/−Nck2iMC* mice (Supplementary Fig. 10c). After *Nck2* deletion at P12–P17, the *Nck1−/−Nck2iMC* mice showed reduced NVT formation, whereas revascularization into the vaso-obliterated area was enhanced compared to the control littermate mice at P17 (Fig. 8a–c). qPCR analysis of the P15 OIR retinas revealed no difference in the expression of *Vegf-a*, *Pdgf-b* and pericyte, and SMC genes in the *Nck1−/−Nck2iMC* mice (Supplementary Fig. 10d). By immunostaining, we detected similar numbers of PDGFRβ+ cells covering vessels in the *Nck1−/−Nck2iMC* compared to control littermate mice, whereas α-SMA staining revealed a 40% decrease of activated pericyte recruitment on the NVTs of *Nck1−/−Nck2iMC* mice (Fig. 8d). Moreover, *Nck1* and *Nck2* deletion decreased DESMIN+ and α-SMA+ mural cell coverage of the angiogenic sprouts in the OIR retina of *Nck1−/−Nck2iMC* mice when compared to control littermates (Fig. 8e, f and Supplementary Fig. 10e). The diameter and SMC coverage of the arterioles were not affected in the Nck1−/−Nck2iMC compared to littermate control mice. However, loss of NCK1/2 function in pericytes did not affect capillary diameter and strongly decreased the vascular leakage compared to control littermate mice (Fig. 8g,h, Supplementary Fig. 10f).

## Discussion

**Fig. 9:**
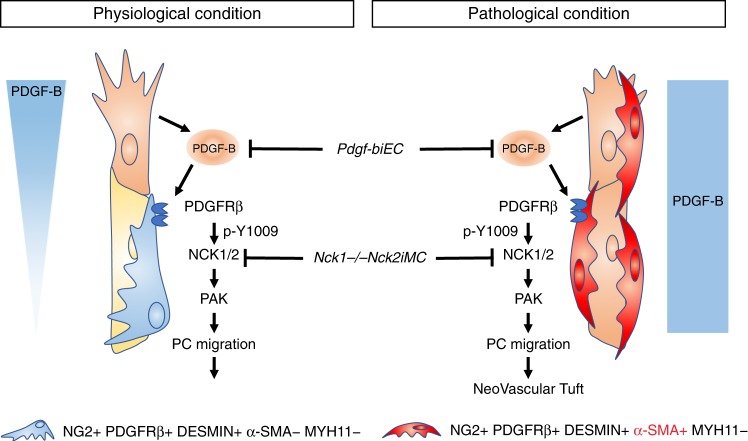
Model for pericyte NCK1/2 function in vascular development and ischemic retinopathy. During angiogenic sprouting, tip cells express PDGF-B to recruit the pericytes and stabilize the new vessels. PDGFRβ activation on the surface of pericytes leads to NCK1/2-dependent PAK activation and pericyte migration. Under ischemic conditions, high PDGF-B levels activate pericyte α-SMA expression and increase vessel coverage, including tip cells. Dysfunctional α-SMA+ pericytes promote pathological angiogenesis and inhibit retinal revascularization. Inhibition of PDGF-B or PDGFRβ downstream NCK1/2 signaling blocks pericyte recruitment and NVTs and promotes vessel regrowth in the OIR retina

This study investigates the role of pericytes and PDGFRβ downstream signaling in a mouse OIR model. Our first main finding is that OIR NVTs and newly sprouting tips are covered with abnormal α-SMA-expressing pericytes. Genetic lineage tracing shows that these cells are pericyte-derived and acquire α-SMA but not MYH11 expression (Fig. 9).

In the normal postnatal retina, we can distinguish pericytes from SMCs by their lack of expression of the contractile protein α-SMA (Fig. 9). Single-cell RNA sequencing confirmed that brain pericytes do not express α-SMA^40^. In fact, this comprehensive analysis showed that brain pericytes do not harbor any unique genes that can be used to distinguish them from SMCs, but are best defined by the presence of mural cell markers such as PDGFRβ and absence of specific SMC genes such as α-SMA, which is in line with our observations.

How acquisition of the contractile protein α-SMA by pericytes contributes to OIR neovascular defects remains to be clarified. We speculate that aberrant positioning around growing angiogenic tip cells, and modification of pericyte contractile properties affecting tip cell extension may play a role. In the normal retina and brain, it is debated whether pericytes, or α-SMA-positive cells at the transition zone between arterioles and capillaries regulate cerebral blood flow (CBF)^41–45^. In brain ischemia models, it has been suggested that ischemic pericytes limit CBF by constricting capillaries, thereby inducing no-reflow^46–48^.

Ischemic OIR retinas up-regulated PDGF-B expression, and ischemia and PDGF-B activated pericyte α-SMA expression in OIR. Loss of endothelial *Pdgf-b* inhibited pericyte α-SMA+ coverage and NVT formation in OIR, indicating that PDGF-B induced pericyte recruitment and dysfunction to promote pathological angiogenesis (Fig. 9). Interestingly, the *Pdgf-biEC* OIR retinas showed residual *Pdgf-b* expression, suggesting the existence of other cell sources of PDGF-B in ischemic retinopathy besides ECs. Ischemic Müller cells and photoreceptors overexpressing *Pdgf-b* in the OIR model might constitute such sources^34,35^. In contrast to OIR, in the normal brain and retina ECs do represent the major source of PDGF-B, as shown previously and recently confirmed by single-cell RNA sequencing^40^. Postnatal genetic deletion of endothelial *Pdgf-b* was sufficient to induce near-complete absence of retinal pericytes, as shown here and in another recent study^19^, confirming endothelial PDGF-B as the major source driving pericyte recruitment in this tissue.

Pericytes are required to maintain functional BRB and retinal vasculature homeostasis^19,36^. Despite the reduced NVT formation in *Pdgf-biEC* mutant OIR mice, these mice also exhibited severe loss of pericyte coverage and vascular defects, including microaneurysms, impaired revascularization, and hemorrhage. NVTs covered with abnormal α-SMA+ pericytes displayed FIBRINOGEN leakage, indicating breakdown of the BRB. Together with increased vascular permeability, the activated state of pericytes during retinopathy might contribute to the loss of BRB. Two recent studies revealed that the loss of the *Foxc1* and *Foxf2* transcription factors increased brain pericyte density but induced BBB defects^49,50^, suggesting that pericyte identity is important for BBB integrity. Pericytes maintain the functional BRB and BBB by restraining EC transcytosis in a process involving the lipid transporter MFSD2A^17,19,51–54^. Therefore, loss of PDGFRβ signaling in human retinopathies is expected to induce BRB leakage.

Our second main finding is that NCK1 and NCK2 signaling downstream of PDGFRβ promotes PDGF-B-induced pericyte migration in vivo and in vitro. P5 *Nck1−/−Nck2iMC* retinas exhibited impaired pericyte recruitment to the sprouting angiogenic front, whereas pericyte attachment and proliferation were similar to controls (Fig. 9). In cultured brain pericytes stimulated with PDGF-B, NCK adapters promoted pericyte migration via PAK activation. NCK1/2 bind PAK, which is directly activated by the small GTPases RAC and CDC42 to regulate cell migration^29,55^. In vivo, NCK-dependent pericyte migration was necessary for vascular sprouting in retinal angiogenesis. Since EC migration is also essential for vessel sprouting^29,56^, pericytes and ECs may coordinate their migration via PDGF-B/NCK signaling to ensure effective angiogenic sprouting and vessel stabilization in vascular development.

Lack of NCK in human and mouse pericytes decreased PDGFRβ Y1009 phosphorylation, whereas it had only a modest impact on PDGFRβ Y751 phosphorylation, which leads to ERK and AKT activation^27^. Likewise, in ECs we have previously shown that NCK1/2 specifically regulates VEGFR2 Y1214 but not Y1175 phosphorylation to promote migration^29,56^. These data suggest the existence of signaling mediators regulated by NCK that affect site-specific phosphorylation of those receptors. Candidates include the ubiquitously expressed tyrosine phosphatase SHP-2 that binds to phosphorylated PDGFRβ Y1009 to regulate cell migration^57^. Additional studies are required to identify these mediators and to elucidate the role of Y1009 in vivo.

In OIR, NCK1/2 deletion inhibited vascular leak and NVT formation, while retinal revascularization was improved. Excessive pericyte coverage of sprouting tips, as well as α-SMA expression were decreased when compared to controls (Fig. 9). NCK1/2-deficient mural cells may hence be able to polarize VEGF-A-induced tip cell sprouting and thereby sustain vessel regrowth in OIR. Altogether, the data suggest that inhibition of NCK1/2-dependent pericyte migration holds promise as a new strategy to treat retinopathies. Using elegant in vivo and biochemical studies, Borroto et al.^58^ have developed selective inhibitors of the TCR–NCK interaction for treating human autoimmune and inflammatory diseases. Examination of PDGFB/PDGFRβ and NCK pathway activation status in patients will determine if specific PDGFRβ Y1009-NCK1/2 inhibitors could be developed for intraocular treatment of ischemic retinopathy.

In summary, this study shows that hypoxia creates a vascular niche for pericyte activation in the ischemic retina. Activated pericytes expressing α-SMA promote pathological angiogenesis and inhibit retinal revascularization in OIR, confirming that pericyte identity and function are essential for vessel sprouting. Controlling pericyte identity might have implications for improving vessel permeability, oxygen delivery, neuronal survival, and vascular malformations in retinopathy. Targeting PDGFRβ-NCK signaling inhibits pathological OIR angiogenesis and promotes tissue revascularization and could be a novel approach to prevent ocular neovascular diseases and improve retinal wound healing.

## Methods

### Mice

Mice were maintained in the Animal Research Center and experiments were performed under a protocol approved by the Institutional Animal Care and Use Committee of Yale University. For inducible Cre-mediated recombination, *Nck1−/−;Nck2flox/flox* mice^29^ (129/SVJ), *Pdgf-bflox/flox mice*^32^ (C57BL/6J) and mTmG reporter mice (The Jackson Laboratory, 129/SVJ) were bred with *Cdh5-CreERT2* mice^59^ (C57BL/6J), *Pdgfrβ-CreERT2* mice^60,61^ (129/SV;C57BL/6J mixed background), *SMACreERT2* mice^62^ (or *ACTA2-CreERT2*, FVB/NJ), and *Myh11CreERT2* mice (The Jackson Laboratory, FVB/NJ). Gene deletion was induced by intraperitoneal injections with 50 μg tamoxifen (Sigma, T5648; 1 mg/ml) to pups at P0, P1, and P2, and mice were sacrificed at P5. The Cre-negative, tamoxifen-treated littermates are used as control mice. For genetic cell fate tracking, CRE activity was induced by two consecutive intraperitoneal injections of pups at P6 and P7 with 50 μg tamoxifen solution.

### Reagents and antibodies

PDGF-BB (220-BB, R&D Systems). Antibodies used were as follows: anti-NCK1 (1/200, ab168940, Abcam), anti-NCK2 (1/200, ab109239, Abcam), anti-PDGF-B (1/50, AB23914, Abcam), anti-PDGFRβ (1/100, AF1042, R&D Systems), anti-NG2 (1/200, AB5320, Millipore), anti-MYH11 (1/200, AB53219, Abcam), anti-DESMIN (1/200, AF3844, R&D Systems), anti-TER119 eFluor 450 (1/50, 48-5921-82, eBioscience-ThermoFisher Scientific), anti-fibronectin (1/300, F3648, Sigma-Aldrich), anti-laminin (1/300, AB11575, Abcam), anti-collagen IV (1/300, AB769, Millipore), anti-GFP (1/300, G10362, Life Technologies), anti-ERG1/2/3 (1/100, SC353, Santa Cruz), anti-phospho-histone 3 (1/100, 06-570, Millipore), anti-α-smooth muscle actin CY3 (1/200, α-SMA, C6198, Sigma), anti-PDGFRβ 751 (1/1000, #4549, Cell Signaling), anti-PDGFRβ 1009 (1/1000, #3124, Cell Signaling), anti-PDGFRβ 1021 (1/1000, #2227, Cell Signaling), anti-PDGFRβ (1/1000, #3169, Cell Signaling), anti-p44/42 MAP kinase (1/1000, phospho-ERK, #9106, Cell Signaling), anti-44/42 MAP kinase (1/1000, total ERK, #9102, Cell Signaling), anti-pAKT (1/1000, #4060, Cell Signaling), and anti-panAKT (1/1000, total AKT, #4685, Cell Signaling). Appropriate secondary antibodies were conjugated to horseradish peroxidase (Vector Laboratories) or fluorescently labeled (Life Technologies). IB4 was purchased from Life Technologies.

### Oxygen-induced retinopathy

The mother and P7 pups were placed in 75% O_2_ until P12. Next, the pups were injected with 400 µg of tamoxifen at P12 and P13 and placed with a nursing mother for adoption. Eyes were collected at P17, retinas were stained with IB4, and avascular area and the tufts were quantified^29,56^. The Cre-negative, tamoxifen-treated littermates were used as control mice. Neither *Pdgf-bl/l* nor *Nck1−/−Nck2l/l* mice carry the rb8 mutation^63^ and their avascular and NVT areas were similar at P17, suggesting that both strains are comparable (Supplementary Fig. 10g).

### Immunohistochemistry

The eyes of P5 pups were prefixed in 4% paraformaldehyde for 20 min at room temperature (RT). The retinas were dissected out and blocked during 30 min at RT in blocking buffer (BB: 1% fetal bovine serum (FBS), 3% bovine serum albumin, 0.5% Triton X-100, 0.01% Na deoxycholate, 0.02% Na azide in phosphate-buffered saline at pH 7.4). The retinas were incubated with antibodies in BB overnight. After washing, the retinas were incubated with IB4 and the corresponding secondary antibody for 2 h at RT. Then, the retinas were mounted in fluorescent mounting medium (Dako, Carpinteria, CA, USA). High-resolution pictures were acquired using a Leica SP5 confocal microscope with a Leica spectral detection system (Leica 15 SP detector) and the Leica application suite advanced fluorescence (LAS-AF) software. Quantification of staining and retinal vascular development was done using the ImageJ software.

α-SMA, DESMIN, NG2, and PDGFRβ coverage was determined by quantifying the α-SMA+ or DESMIN+ or NG2+ or PDGFRβ+ area normalized to IB4+ area in ×63 images. The RBC leakage was measured by quantifying the number of extravascular TER119+ cells normalized to the total number of TER119+ cells in ×63 images. FIBRINOGEN deposition was measured by quantifying the FIBRINOGEN area normalized to IB4+ area in ×20 images. The pups used for FIBRINOGEN staining were not perfused.

### Cell culture and siRNA transfection

HBVPs (#1200) and human brain microvascular endothelial cells (HBMECs, #1000) were obtained from ScienCell^TM^ and cultured on 0.1% gelatin-coated plates in EGM2 medium (Lonza). These conditions promote pericyte marker expression and inhibit SMC marker expression^38^. Human brain vascular SMCs (#1100) were obtained from ScienCell^TM^ and cultured on 0.1% gelatin-coated plates in Dulbecco's modified Eagle's medium (Gibco by Life Technologies, #10569-010) supplemented with 10% FBS and 1% of penicillin/streptomycin solution and used for experiments until passage 4. HBVPs and HBMECs were maintained in EGM2 and used for experiments until passages 5 and 4, respectively. HBVPs were starved overnight in EBM2 supplemented with 2% FBS and treated with PDGF-b during the time indicated. siRNAs (FlexiTube siRNA) were purchased from Dharmacon (Supplementary Table 1), NCK1 (SMARTpool: ON-TARGETplus NCK1 siRNA L-006354-00-0005), NCK2 (SMARTpool: ON-TARGETplus NCK2 siRNA L-019547-00-0005), PDGFRβ (SMARTpool: ON-TARGETplus PDGFRb L-003163-00-0005), and the negative control (ON-TARGETplus Non-targeting Pool D-001810-10-05). During the transfection, the cells were cultured in Opti-MEM^®^ I Reduced Serum Medium (Cat. No. 31985-062) and the HBVPs were transfected with 25 pmol siRNA and 2 μl RNAiMax (Invitrogen) per well in 6-well plates, according to the manufacturer’s instructions. The medium of the cells was changed 4 h after transfection and the HBVPs were cultured in EGM2 up to 48 h.

### Scratch assay

Confluent HBVP monolayers were grown in 6-well plates. Forty-eight hours after siRNA transfection, a horizontal wound was created using a sterile 200 µl pipette tip. Next, the cells were washed with EBM2 at 37 °C and incubated in EGM2 supplemented with PDGF-B (25 ng/ml) at 37 °C for 6 h. Pictures of scratch wounds were taken just before stimulation (time 0) and after 6 h. Migration was calculated using the ImageJ software.

### Proliferation assay

The xCELLigence RTCA DP analyzer was used to measure proliferation of control, *PDGFRβ* and *NCK1/2* knockdown HBVP cells (10,000 cells per well) in response to PDGF-B (100 ng/ml). The plate was monitored every 5 h for 48 h^29^.

### Western blotting

After overnight starvation in EBM2 supplemented with 2% FBS, the cells were stimulated with PDGF-B (20 ng/ml) during the time indicated. Cells were lysed in Laemmli buffer including phosphatase and protease inhibitors (Thermo Scientific, 78420, 1862209). Fifteen micrograms of proteins were separated on 4–15% Criterion precast gel (#567-1084, Bio-Rad) and transferred on nitrocellulose membrane (Bio-Rad). Western blots were developed on a Luminescent image analyzer, ImageQuant LAS 4000 mini (GE Healthcare). See Supplementary Figs. 11 and 12 for the uncropped immunoblots.

### Quantitative real-time PCR

RNAs from HBVP or from retina were purified using RNeasy Kit (Qiagen). One microgram of RNA was reverse transcribed using SuperScript III (Invitrogen) and quantitative PCR were assayed using the corresponding primers (Qiagen, Supplementary Table 2). The expression levels were normalized to HsACTB, MmActb or HsGAPDH, and MmGapdh.

### Statistical analysis

No statistical methods were used to determine sample size before experiments. No randomization and blinding were used. All data are shown as mean ± standard error of the mean (SEM). Samples with equal variances were tested using Mann–Whitney *U* test or two-tailed Student’s *t* test between groups. *P* value 0.05 was considered to be statistically significant. Statistical analyses were performed for all quantitative data using Prism 6.0 (GraphPad).

### Data availability

All data that support the findings of this study are available within the article and its Supplementary Information File and from the corresponding author upon reasonable request.

## Electronic supplementary material

Supplementary Information
