# Supplementary material for: NCK-dependent pericyte migration promotes pathological neovascularization in ischemic retinopathy
Source: Nat Commun. 2018 Aug 27;9:3463. doi: 10.1038/s41467-018-05926-7 (PMC6110853; doi:10.1038/s41467-018-05926-7)
Supplement: Supplementary file 1 — Supplementary Information [file 41467_2018_5926_MOESM1_ESM.pdf]

NCK-dependent pericyte migration promotes pathological neovascularization in ischemic retinopathy

Dubrac et al.

Supplementary Figure 1

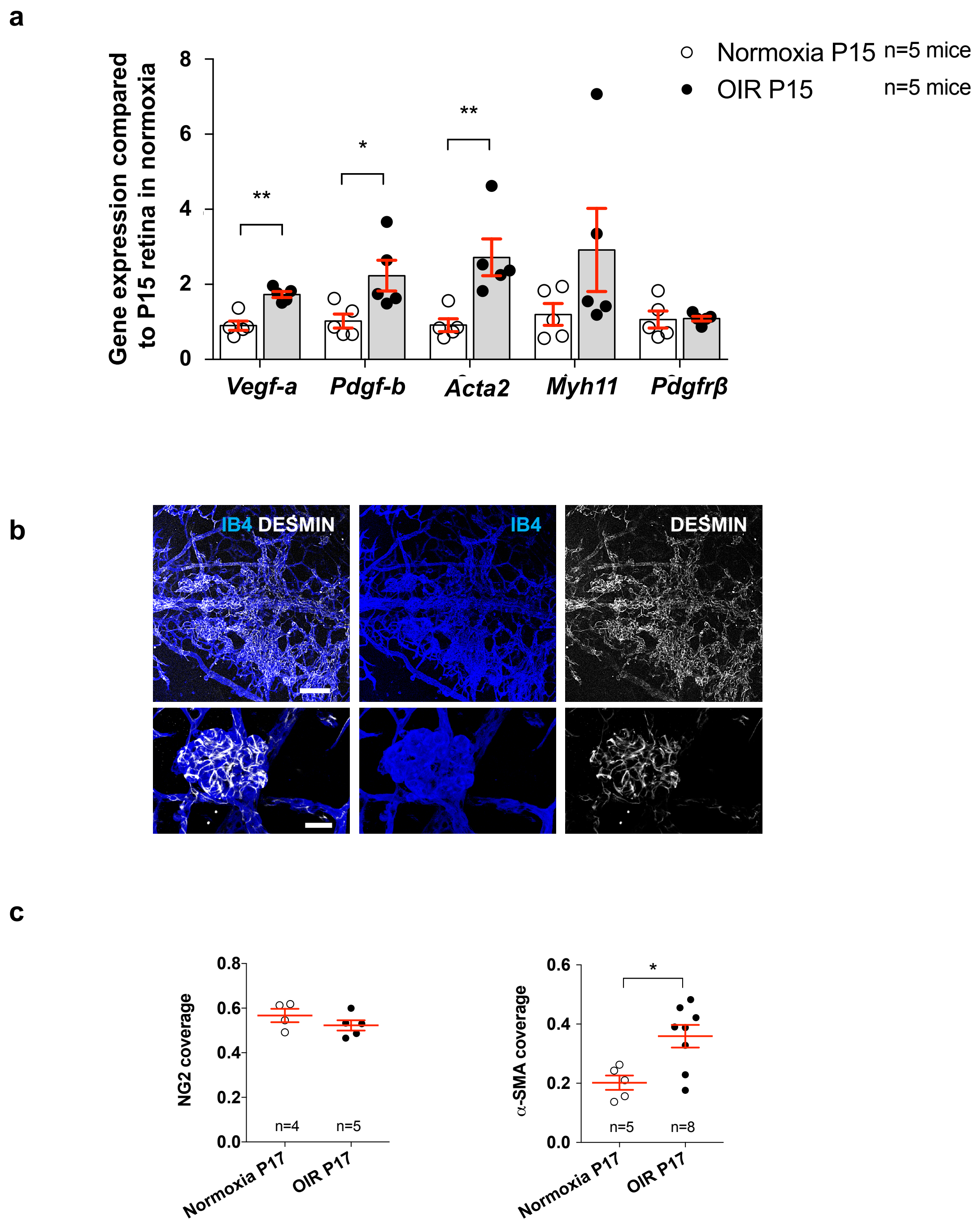

Supplementary Figure 1: Mural cell characterization of OIR retina. (**a**) qPCR analysis of *Vegf-a, Pdgf-b, Acta2, Myh11* and *Pdgfr*β expression in P15 OIR retinas compared to P15 retinas in normoxia. Number of mice used for quantification is indicated. Error bars: s.e.m. * P<0.05, Mann–Whitney U test; ** P<0.01, Mann–Whitney U test. (**b**) IB4 and DESMIN double staining of P17 OIR retinas. Scale bars, top 100 μm and bottom 30 μm. (**c**) Quantification NG2 or α-SMA coverage of P17 retina in normoxia and OIR. Number of retinas used for quantification is indicated. Error bars represent s.e.m. * P<0.05, Mann–Whitney U test.

Supplementary Figure 2

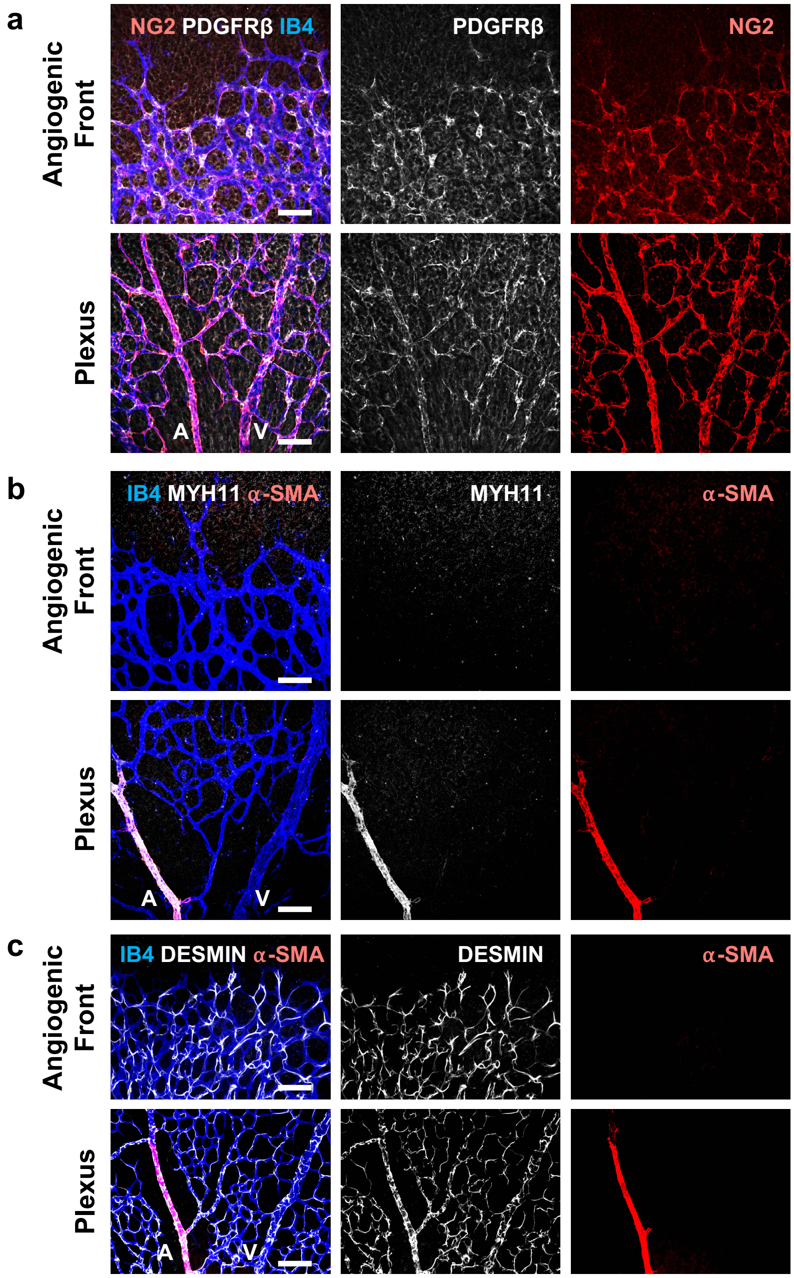

Supplementary Figure 2: Mural cell characterization of developing and OIR retina. (**a**) IB4, NG2 and PDGFRβ triple staining of P5 retina. Scale bars, 100 μm. (**b**) IB4, α-SMA and MYH11 triple staining of P5 retina. Scale bars, 100 μm. (**c**) IB4, DESMIN and α-SMA triple staining of P5 retina. Scale bars, 200 μm

Supplementary Figure 3

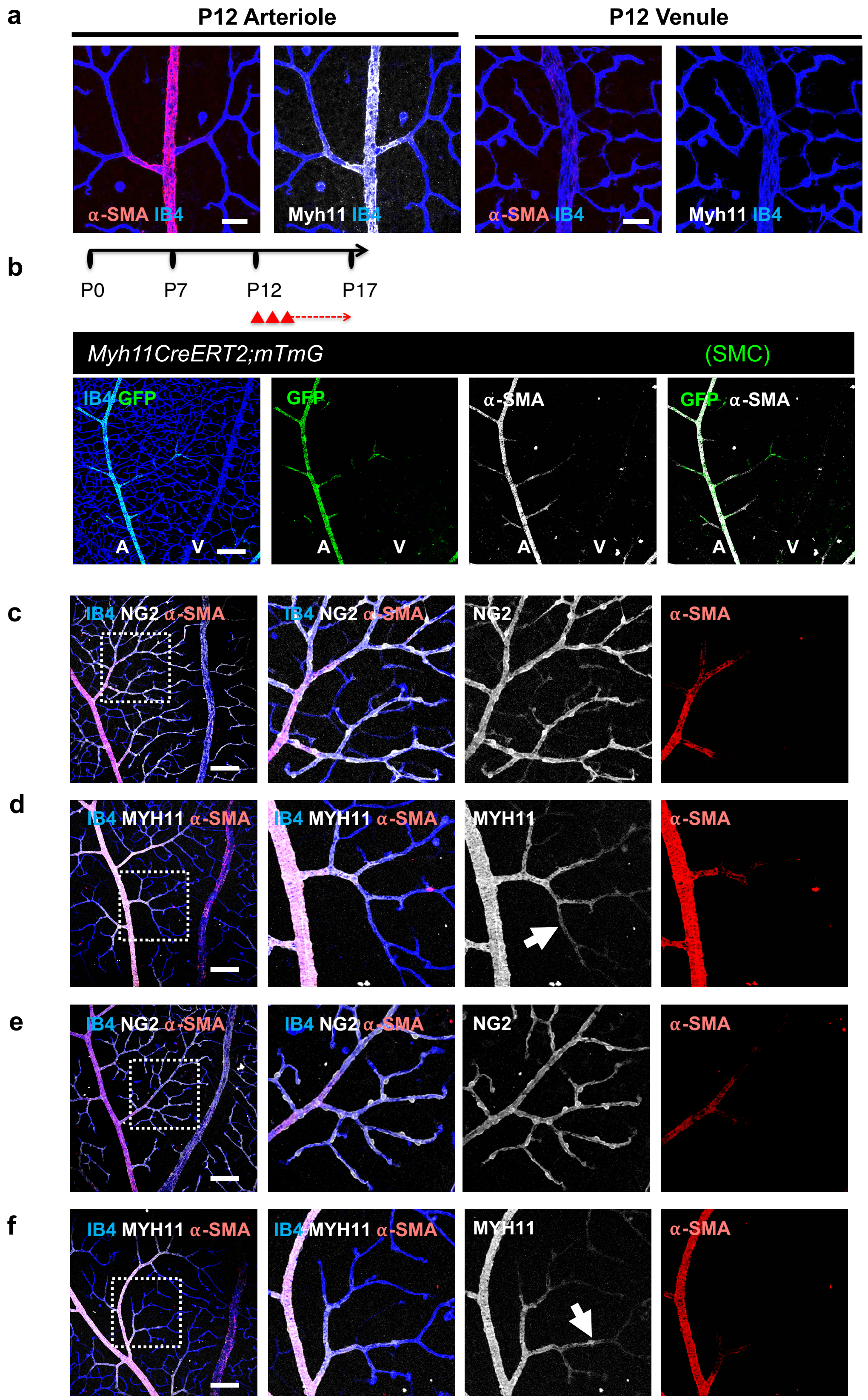

Supplementary Figure 3: Mural cell characterization of postnatal and adult retina. (**a**) IB4, α-SMA and MYH11 triple staining of P12 retina. Scale bars, 30 μm. (**b**) Top: Schematic showing the strategy for genetic labelling of MYH11+ cells by tamoxifen treatment (red triangle) in the corresponding mice. Bottom: IB4, α-SMA and GFP triple staining of P17 retina. Scale bar, 200 μm. (**c-f**) IB4, NG2 and α-SMA triple staining (**c, e**) or IB4, α-SMA and MYH11 triple staining (**d, f**) of P17 retina (**c, d**) and P25 retina (**e, f**). Scale bars, 100 μm. White arrows show MYH+ pericytes.

Supplementary Figure 4

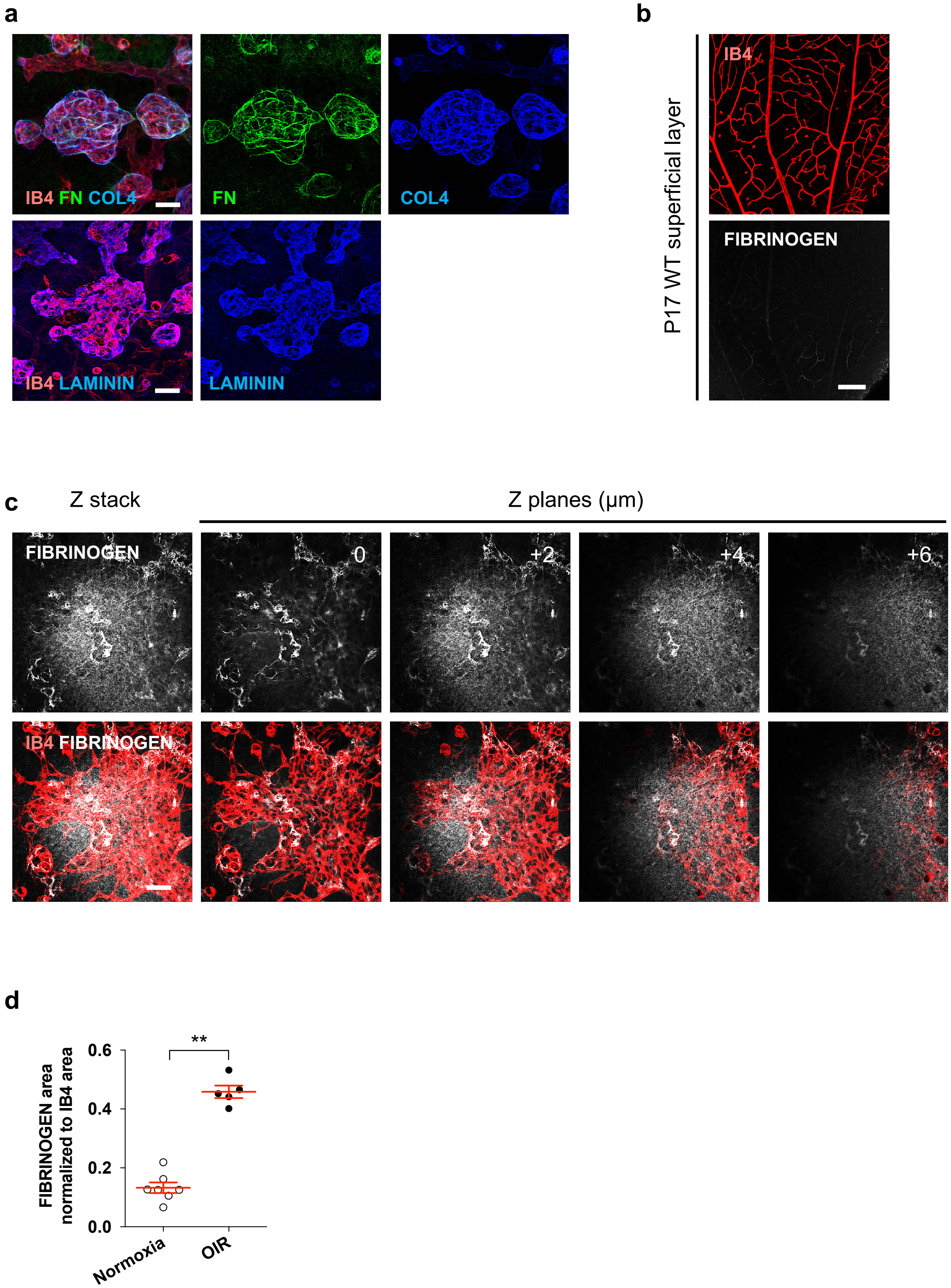

Supplementary Figure 4: Extracellular matrix and FIBRINOGEN staining of OIR retina. (**a**) Top, IB4, FIBRONECTIN (FN) and COLLAGEN 4 (Col4) triple staining of vascular tufts from P17 OIR retinas. Bottom, IB4 and Laminin double staining of vascular tufts. Scale bars, 30 μm. (**b-c**) IB4 and FIBRINOGEN double staining of P17 retinas in normoxia (b) and in OIR (c). Scale bars, 100 μm (b) and 30 μm (c). (**d**) quantification of FIBRINOGEN leakage of P17 retinas in normoxia (n=7) and in OIR (n=5). Error bars represent s.e.m. ** P<0.01, Mann–Whitney U test.

Supplementary Figure 5

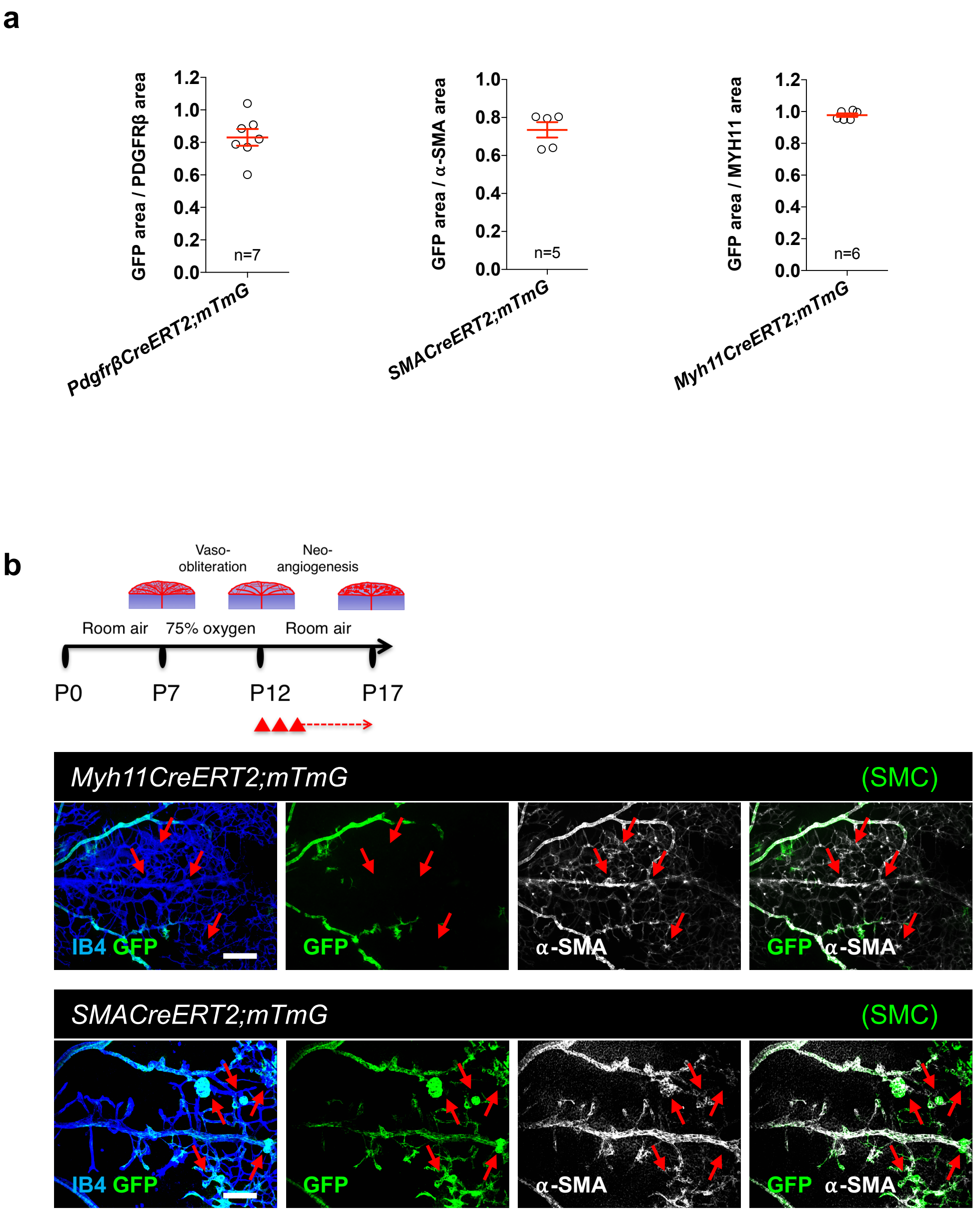

Supplementary Figure 5: MYH11+ α-SMA+ mural cell patterning during development and OIR. (**a**) Quantification of CRE recombination efficiency. Number of retinas used for quantification is indicated. (**b**) Top: Schematic showing the strategy for genetic labelling of MYH11+ or α-SMA cells by tamoxifen treatment (red triangle) in the corresponding mice. Bottom: IB4, α-SMA and GFP triple staining of P17 retina from *Myh11CreERT2;mTmG* or *SMACreERT2;mTmG* mice. Red arrows show α-SMA+ NVTs. Scale bars, 100 μm.

Supplementary Figure 6

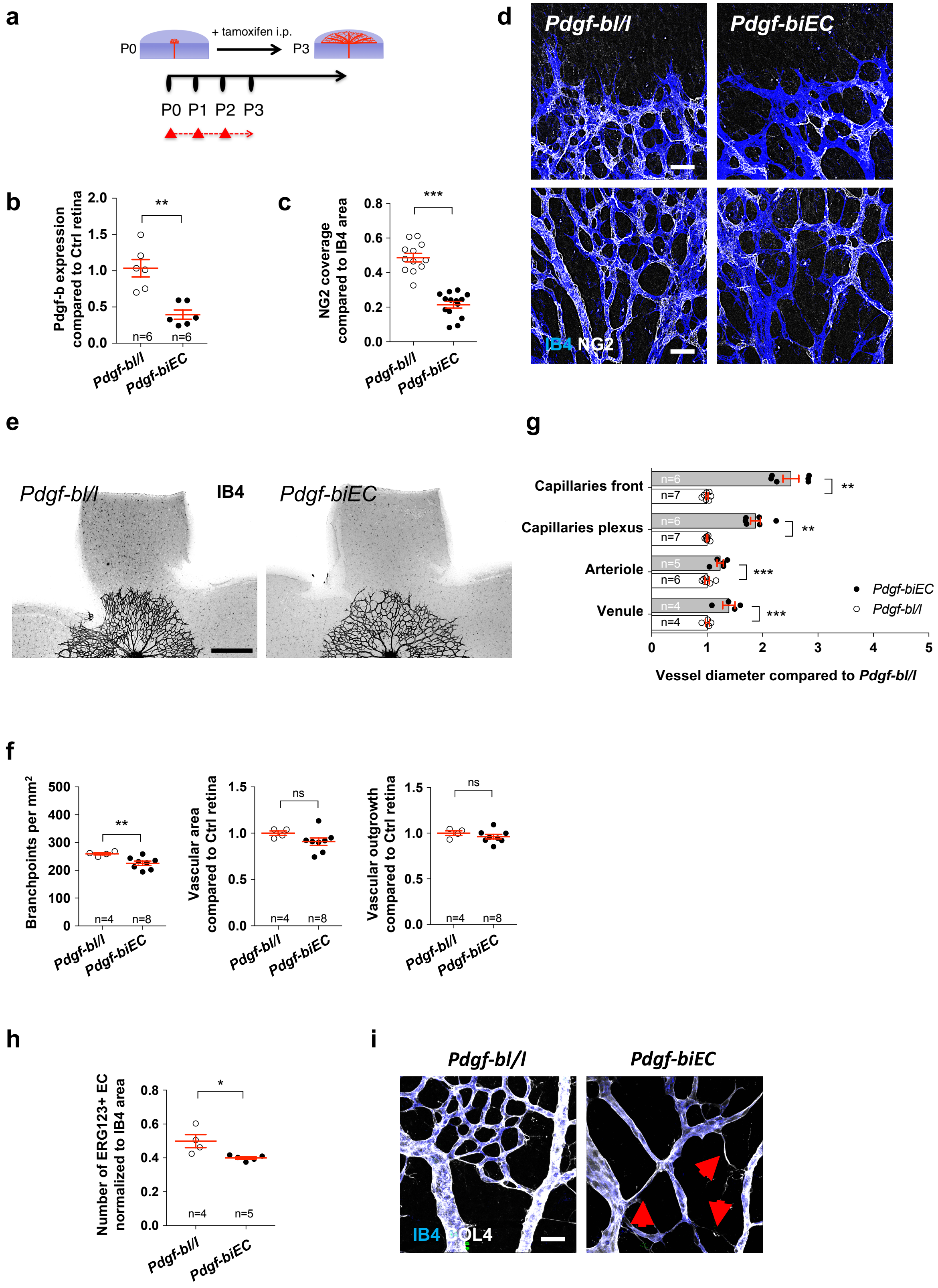

Supplementary Figure 6: Postnatal endothelial *Pdgf-b* deletion decreases pericyte coverage prior to effects on vascularization. (**a**) Schematic of the experimental strategy to assess early formation of the retinal vasculature (P0–P3). The red triangles indicate the intraperitoneal injections of tamoxifen at P0/1/2. (**b**) qPCR measurement of *Pdgf-b* levels in retina from mice with the indicated genotypes. Number of retinas used for quantification is indicated. Error bars represent s.e.m. ** P<0.01, Mann–Whitney U test. (**c**) Quantification of NG2 coverage of IB4+ vessels of P3 retina (n=12 imaging fields for *Pdgf-bl/l* from 6 retinas and n=14 for *Pdgf-biEC* from 6 retinas). Error bars represent s.e.m. *** P<0.001, Mann–Whitney U test. (**d**) IB4 and NG2 double staining of the angiogenic front and the plexus of P3 retina. Scale bars, 30 μm. (**e**) IB4 staining of retinal flat mounts of P3 mice with the indicated genotypes (negative images of the fluorescent signal). Scale bar, 500 μm. (**f**) Quantification of branch points, vascular area and vascular outgrowth. Number of retinas used for quantification is indicated. Error bars represent s.e.m. n.s.: non-significant. ** P<0.01, Mann–Whitney U test. (**g**) Quantification of vessel diameter of P5 retinas. Number of retinas used for quantification is indicated. Error bars represent s.e.m. ** P<0.01, *** P<0.001, Mann–Whitney U test. (**h**) Quantification the ERG1/2/3 positive EC in the vascular front of P5 retinas normalized to vascular area. Number of retinas used for quantification is indicated. Error bars represent s.e.m. * P<0.05, Mann–Whitney U test. (**i**) IB4 and Collagen 4 (Col4) double staining of P5 retina. Red arrows show empty BM sleeves of retracting vessels. Scale bar, 30 μm.

Supplementary Figure 7

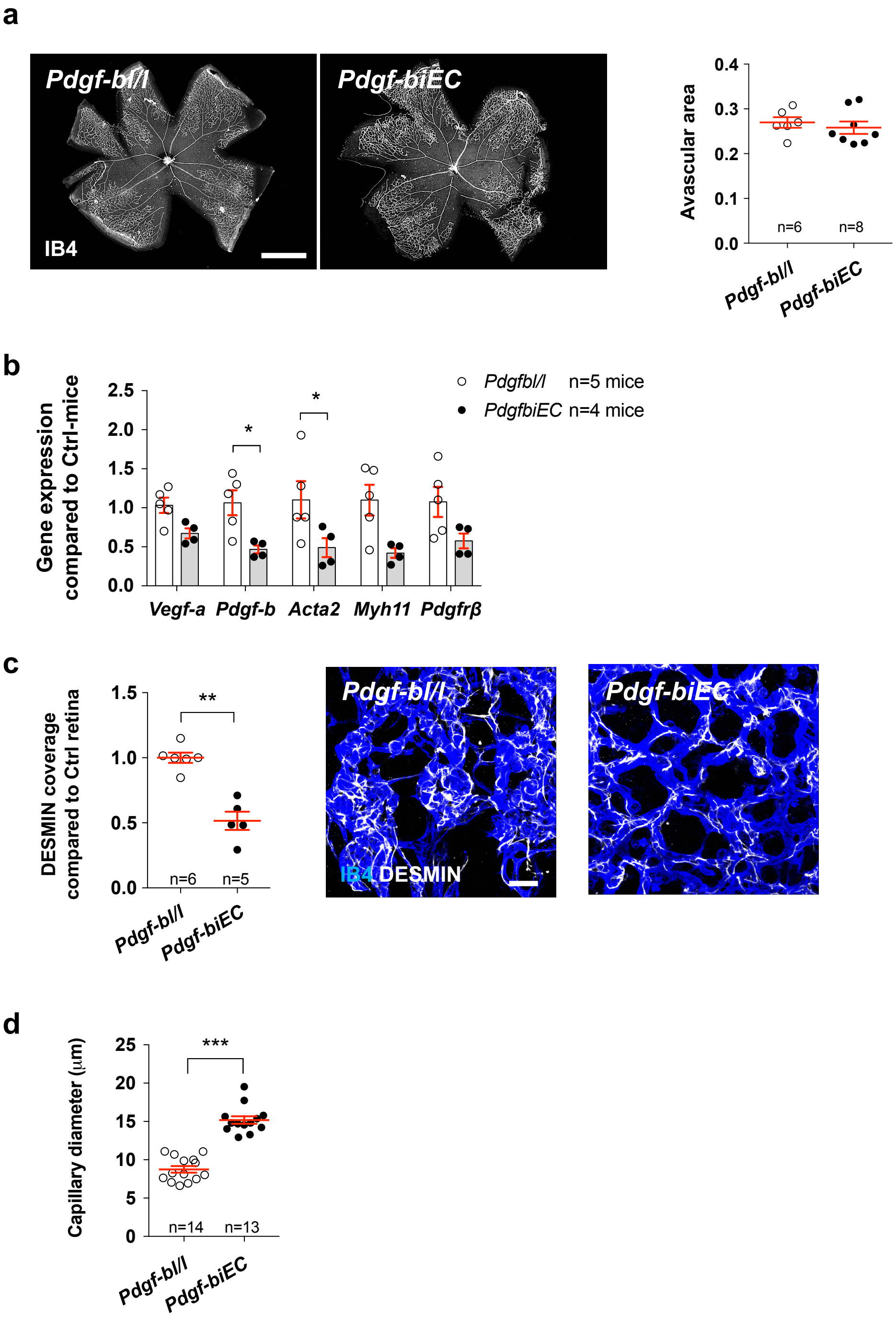

Supplementary Figure 7: Endothelial PDGF-B is essential for pericyte coverage during OIR. (**a**) IB4 staining of retinal flat mounts of P12 mice with the indicated genotypes after hyperoxia and avascular area quantification. Scale bar, 1 mm. Number of retinas used for quantification is indicated. (**b**) qPCR analysis of *Vegf-a, Pdgf-b, Acta2, Myh11* and *Pdgfrβ* expression in P15 OIR retinas compared to control littermate mice. Error bars: s.e.m. * P<0.05, Mann–Whitney U test. (**c**) Left, quantification of DESMIN coverage of IB4+ vascular tufts. Right, IB4 and DESMIN double staining of the NVTs. Scale bar, 30 μm. Number of retinas used for quantification is indicated. Error bars represent s.e.m. ** P<0.01, Mann–Whitney U test. (**d**) Quantification of capillary diameter of P17 retinas after OIR. Number of retinas used for quantification is indicated. Error bars represent s.e.m. *** P<0.001, Mann–Whitney U test.

Supplementary Figure 8

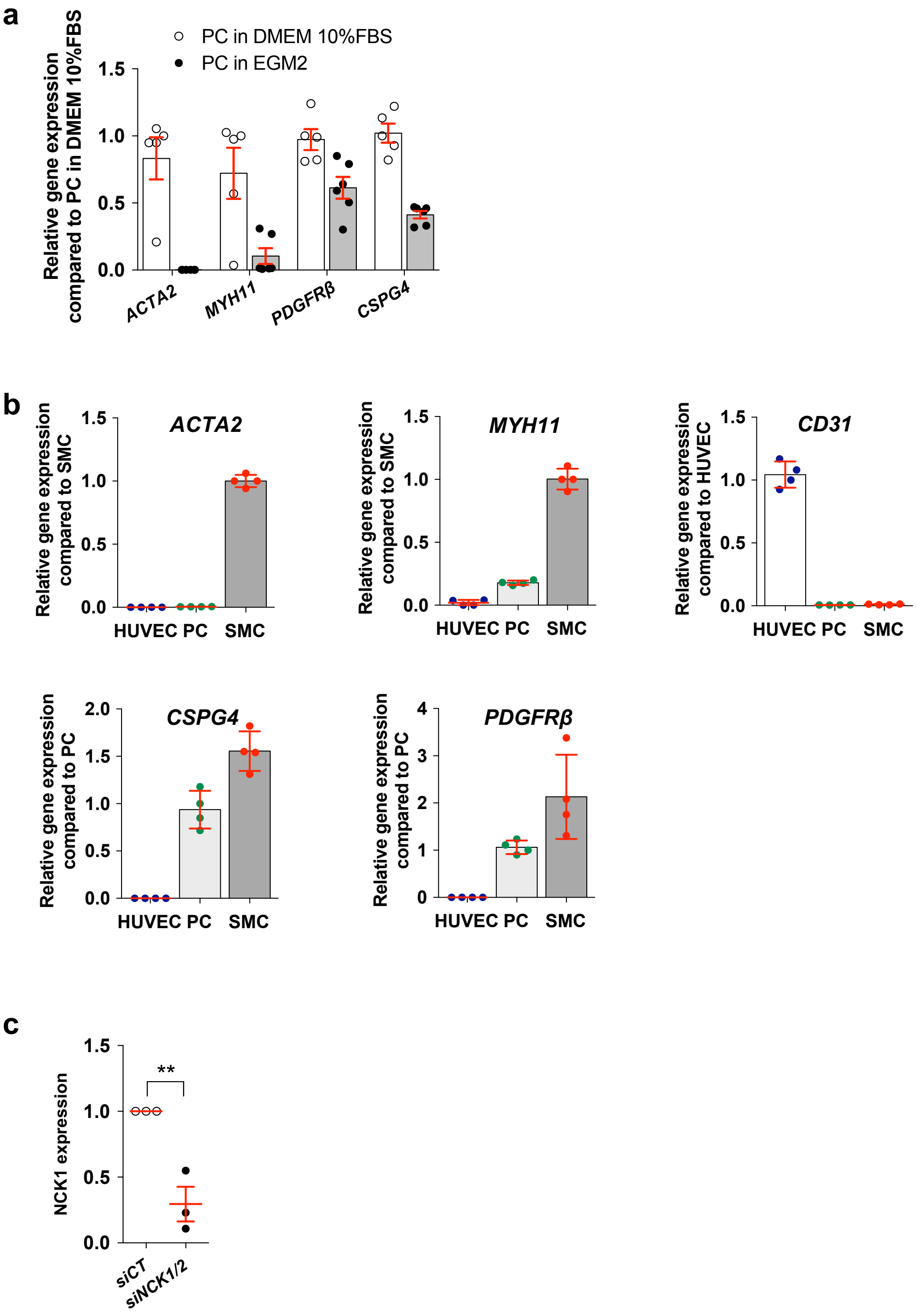

Supplementary Figure 8: Pericyte characterization in vitro. (**a**) qPCR analysis of *ACTA2 (α-SMA)*, *MYH11*, *PDGFRβ and CSPG4 (NG2)* expression in HBPV cells growth with the indicated medium (n = 5 and 6 independent experiments). (**b**) qPCR analysis of *CD31*, *ACTA2 (α-SMA)*, *MYH11*, *PDGFRβ and CSPG4 (NG2)* expression in HBPVC grown in EGM2 compared to SMC and HUVEC (n = 4 independent experiments). (**c**) NCK1 protein expression quantification in HBPV cells treated with siRNA control or against NCK1&2 (corresponding to Western-blot Figure 5**g,** n = 3). Error bars represent s.e.m. ** $P<0.01$, Student's t-test.

Supplementary Figure 9

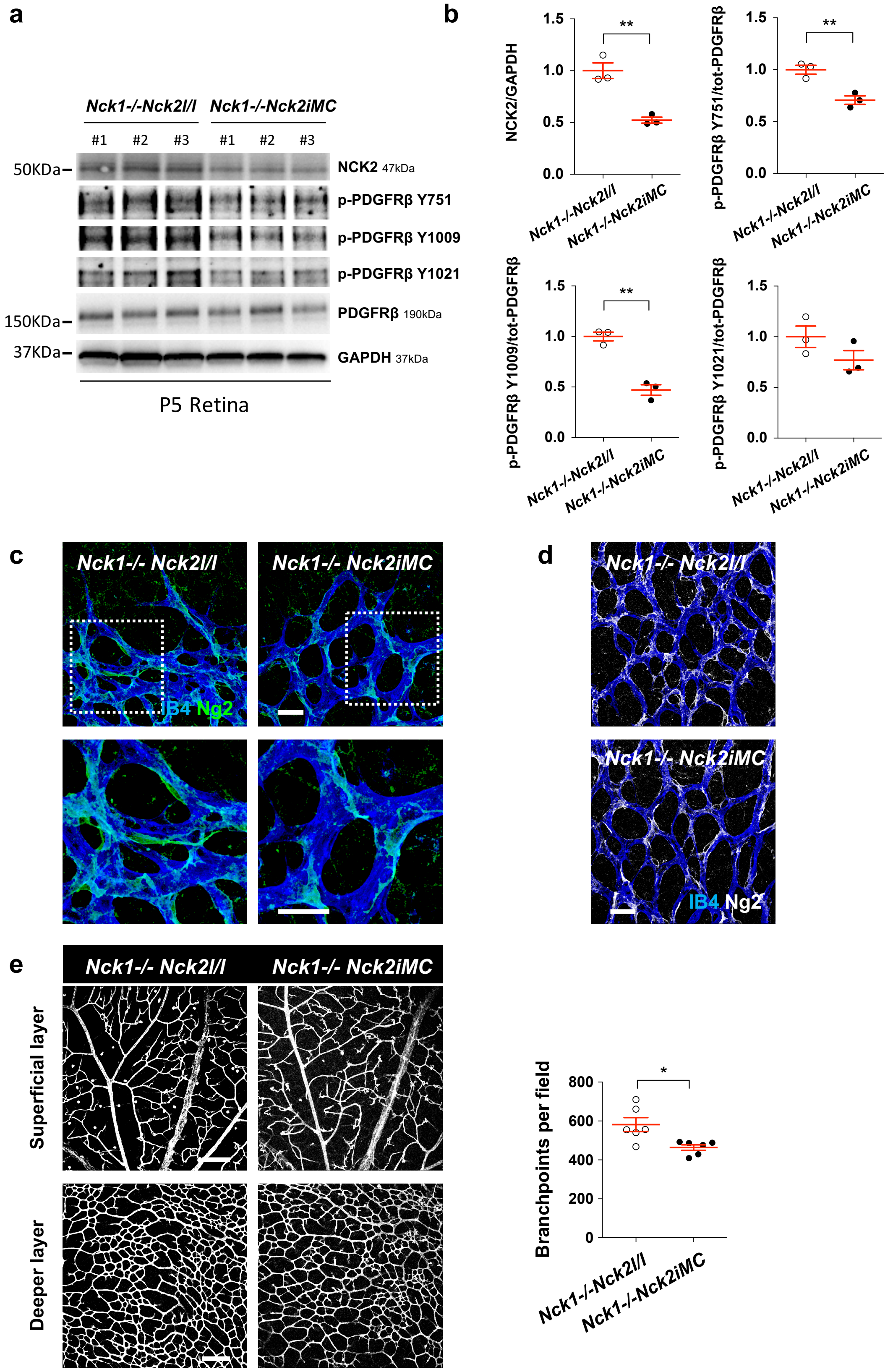

Supplementary Figure 9: Pericyte NCK deletion decreases Pdgfrβ phosphorylation and migration. (**a**) Western-blot using retina from mice with the indicated genotype. (**b**) Quantification (n = 3 independent experiments). Error bars represent s.e.m. ** $P<0.01$, Student's t-test. (**c**) Top: IB4 and NG2 double staining of the angiogenic front of P5 retina of mice with the indicated genotype. Bottom: magnified area of boxed area in the top pictures. Scale bars, 30 μm. (**d**) IB4/DESMIN double-staining of the P5 retina plexus of mice with the indicated genotype. Scale bar, 30 μm. (**e**) Left, IB4 staining of the superficial (top) and deeper (bottom) layer of P12 retina of mice with the indicated genotype. Scale bars, 100 μm. Right, quantification of branchpoints of the deeper layer (n = 6 retina). Error bars represent s.e.m. * $P<0.05$, Mann–Whitney U test.

Supplementary Figure 10

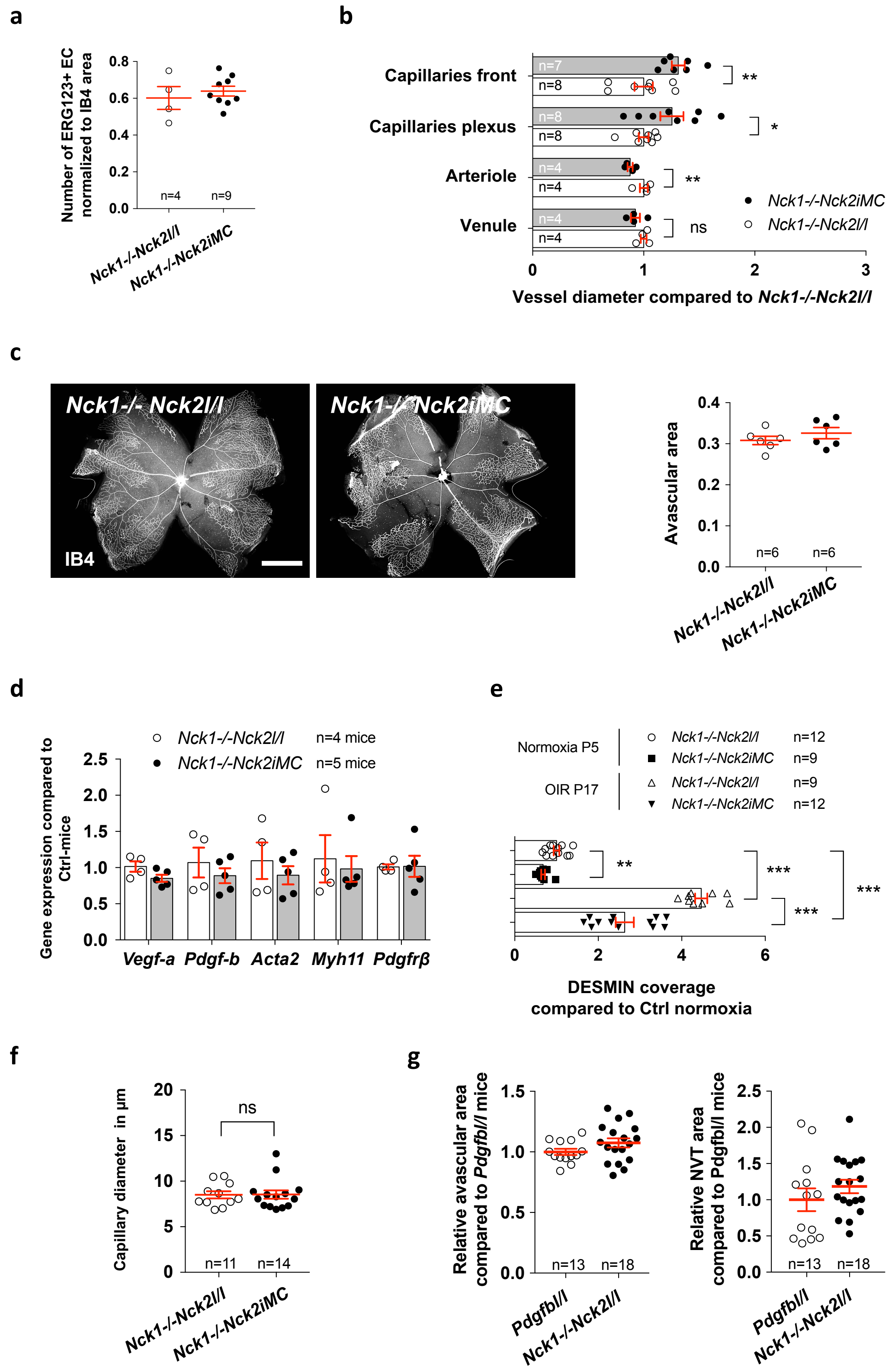

Supplementary Figure 10: NCK deletion does not affect growth factors expression and vessel diameter in OIR. (**a**) Quantification the ERG1/2/3 positive EC in the vascular front of P5 retinas normalized to vascular area. Error bars represent s.e.m. (**b**) Quantification of P5 retina vessel diameter. Number of retinas used for quantification is indicated. Error bars represent s.e.m. * P<0.05, ** P<0.01, Mann–Whitney U test. (**c**) IB4 staining of retinal flat mounts of P12 mice with the indicated genotypes after hyperoxia and avascular area quantification. Scale bar, 1 mm. (**d**) qPCR analysis of *Vegf-a, Pdgf-b, Acta2, Myh11* and *Pdgfr*β expression in P15 OIR retinas compared to control littermate mice. Number of retinas used for quantification is indicated. Error bars: s.e.m. (**e**) Quantification DESMIN coverage in the vascular front of P17 OIR retinas (P5 normoxia: n = 12 imaging fields for *Nck1-/-Nck2l/l* from 8 retinas and n=9 for *Nck1-/-Nck2iMC* from 8 retinas; P17 OIR: n = 9 imaging fields for *Nck1-/-Nck2l/l* from 6 retinas and n=12 for *Nck1-/-Nck2iMC* from 6 retinas). Error bars represent s.e.m. ** P<0.01, *** P<0.001, Mann–Whitney U test. (**f**) Quantification of capillary diameter of P17 retinas after OIR. Number of retinas used for quantification is indicated. Error bars represent s.e.m. n.s. non-significant. (**g**) Quantification of avascular area and NVT area of P17 retinas after OIR. Number of retinas used for quantification is indicated. Error bars represent s.e.m.

Supplementary Figure 11

Uncropped blots for Figure 6g

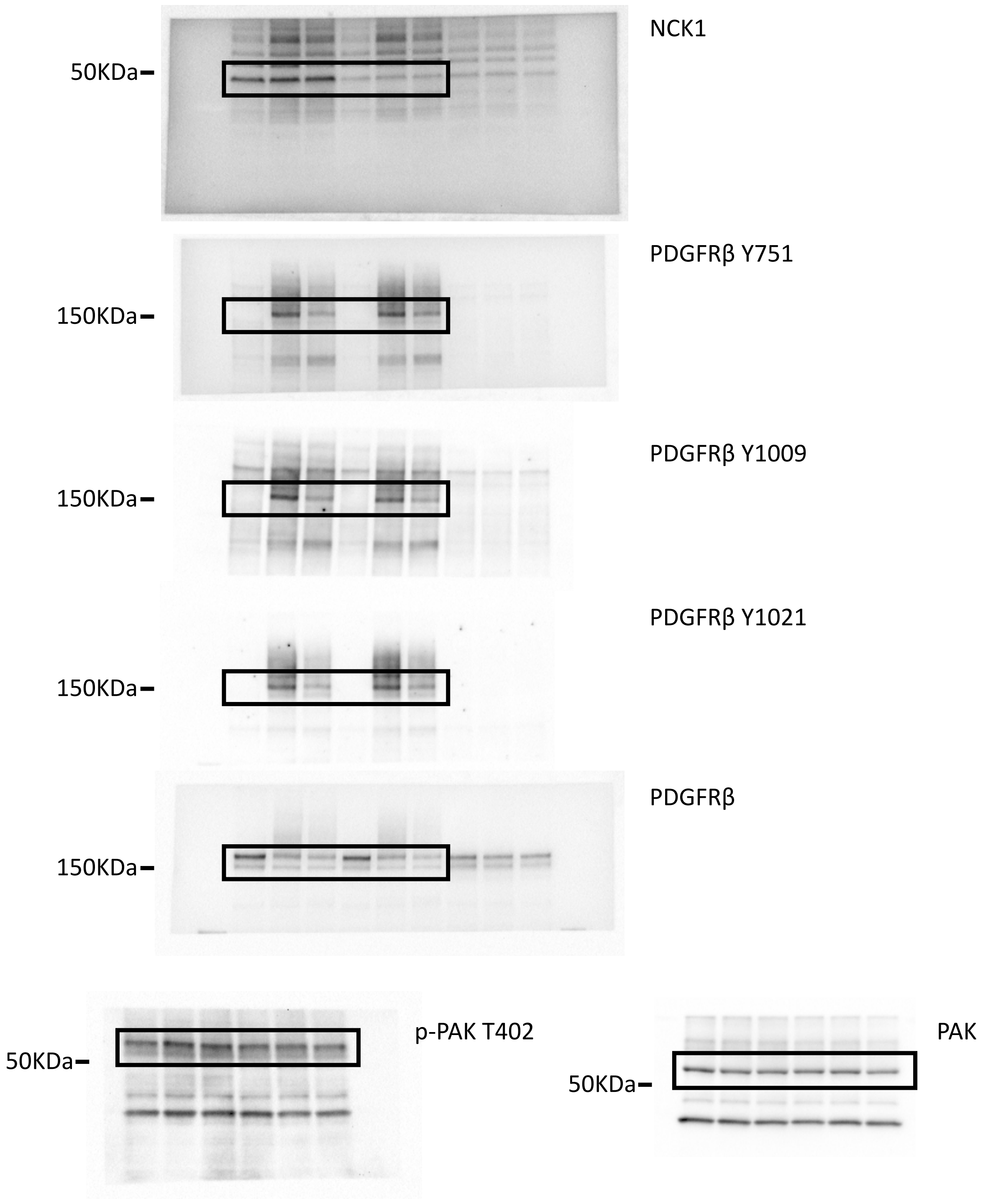

Supplementary Figure 12

Uncropped blots for Figure 6g

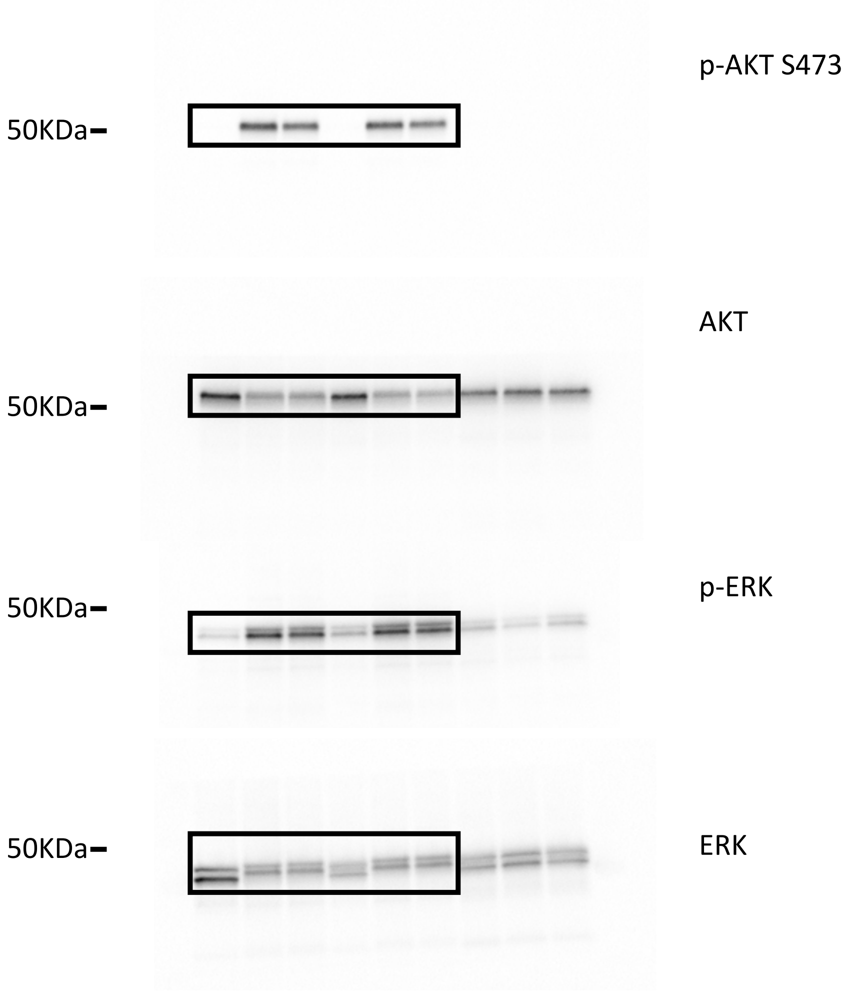

Uncropped blots for Figure 7b

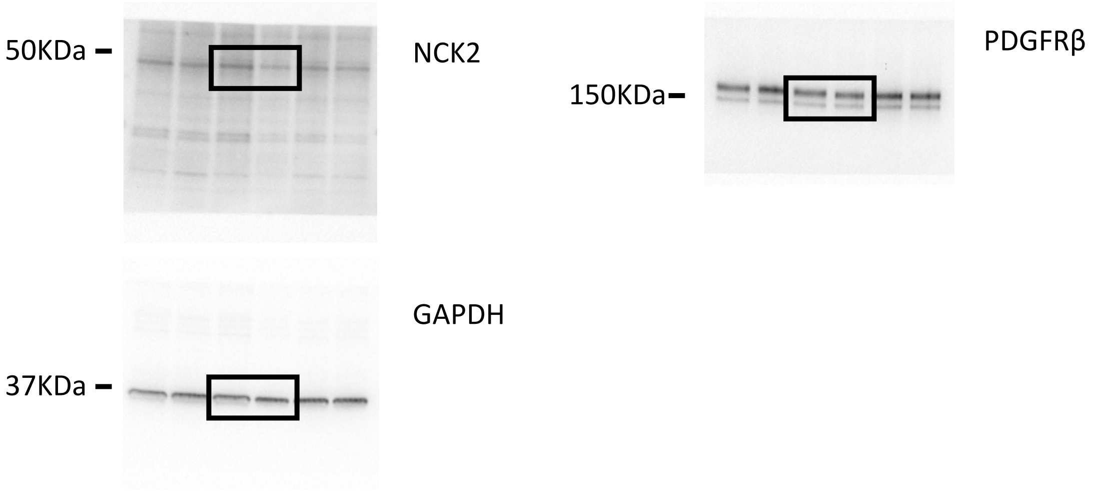

Supplementary Table 1. SiRNA targeting sequences

siRNA	Manufacturer	Sequences
NCK1	Dharmacon	SMARTpool: ON-TARGETplus NCK1 siRNA L-006354-00
NCK2	Dharmacon	SMARTpool: ON-TARGETplus NCK2 siRNA L-019547-00
PDGFRβ	Dharmacon	SMARTpool: ON-TARGETplus PDGFRb L-003163-00
Negative control	Dharmacon	ON-TARGETplus Non-targeting Pool D-001810-10-05

Supplementary Table 2. qPCR primers sequences

Primers	Species reactivity	Sequences
NCK1	Homo sapiens	Qiagen, QT00077945
NCK2	Homo sapiens	Qiagen, QT00245441
PDGFRβ	Homo sapiens	Qiagen, QT00082327
ACTA2	Homo sapiens	Qiagen, QT00088102
MYH11	Homo sapiens	Qiagen, QT00069391
CSPG4	Homo sapiens	Qiagen, QT00079884
ACTB	Homo sapiens	Qiagen, QT01680476
GAPDH	Homo sapiens	Qiagen, QT00079247
Nck1	Mus musculus	Qiagen, QT00134484
Nck2	Mus musculus	Qiagen, QT00142324
Vegfa	Mus musculus	Qiagen, QT00160769
Pdgfb	Mus musculus	Qiagen, QT00266910
Acta2	Mus musculus	Qiagen, QT00140119
Myh11	Mus musculus	Qiagen, PPM04496A
Pdgfrβ	Mus musculus	Qiagen, QT00113148
Cspg4	Mus musculus	Qiagen, QT00120407
Actb	Mus musculus	Qiagen, QT01136772
Gapdh	Mus musculus	Qiagen, QT01658692
